# Processes of behavior change and weight loss in a theory-based weight loss intervention program: a test of the process model for lifestyle behavior change

**DOI:** 10.1186/s12966-014-0160-6

**Published:** 2015-01-16

**Authors:** Fiona Gillison, Afroditi Stathi, Prasuna Reddy, Rachel Perry, Gordon Taylor, Paul Bennett, James Dunbar, Colin Greaves

**Affiliations:** Department for Health, University of Bath, Bath, BA2 7AY UK; School of Medicine and Public Health, University of Newcastle, University Drive, Callaghan, NSW 2308 Australia; Centre for Exercise, Nutrition and Health Sciences, University of Bristol, 8 Priory Road, Bristol, BS8 1TZ UK; Greater Green Triangle University Department of Rural Health, Flinders and Deakin Universities, PO Box 423, Warrnambool, Victoria 3280 Australia; University of Exeter Medical School, St Luke’s Campus, Magdalen Road, Exeter, EX1 2LU UK

**Keywords:** Physical activity, Diet, Weight loss intervention, Process evaluation

## Abstract

**Background:**

Process evaluation is important for improving theories of behavior change and behavioral intervention methods. The present study reports on the process outcomes of a pilot test of the theoretical model (the Process Model for Lifestyle Behavior Change; PMLBC) underpinning an evidence-informed, theory-driven, group-based intervention designed to promote healthy eating and physical activity for people with high cardiovascular risk.

**Methods:**

108 people at high risk of diabetes or heart disease were randomized to a group-based weight management intervention targeting diet and physical activity plus usual care, or to usual care. The intervention comprised nine group based sessions designed to promote motivation, social support, self-regulation and understanding of the behavior change process. Weight loss, diet, physical activity and theoretically defined mediators of change were measured pre-intervention, and after four and 12 months.

**Results:**

The intervention resulted in significant improvements in fiber intake (M between-group difference = 5.7 g/day, p < .001) but not fat consumption (−2.3 g/day, p = 0.13), that were predictive of weight loss at both four months (M between-group difference = −1.98 kg, p < .01; R^2^ = 0.2, p < 0.005), and 12 months (M difference = −1.85 kg, p = 0.1; R^2^ = 0.1, p < 0.01). The intervention was successful in improving the majority of specified mediators of behavior change, and the predicted mechanisms of change specified in the PMBLC were largely supported. Improvements in self-efficacy and understanding of the behavior change process were associated with engagement in coping planning and self-monitoring activities, and successful dietary change at four and 12 months. While participants reported improvements in motivational and social support variables, there was no effect of these, or of the intervention overall, on physical activity.

**Conclusions:**

The data broadly support the theoretical model for supporting some dietary changes, but not for physical activity. Systematic intervention design allowed us to identify where improvements to the intervention may be implemented to promote change in all proposed mediators. More work is needed to explore effective mechanisms within interventions to promote physical activity behavior.

**Electronic supplementary material:**

The online version of this article (doi:10.1186/s12966-014-0160-6) contains supplementary material, which is available to authorized users.

## Background

Cardiovascular disease is a major cause of poor health and wellbeing [1,2]. As obesity and physical inactivity are significant contributors to its development [3,4], interventions aiming to prevent cardiovascular disease commonly target the modifiable lifestyle behaviors of dietary intake and physical activity [5,6]. While some lifestyle interventions show promise in prompting behavior changes sufficient to lead to clinical improvements in similar populations behavior [7-10], there is a wide variation in effectiveness, especially over the longer term [11-13]. It has been argued that our ability to improve behavioral interventions is restricted by a lack of sufficient detail and rigor in intervention design, and inadequate evaluation of the mechanisms of behavior change. Improving this would allow us to build a cumulative science base for lifestyle behavior change [14-16]. Process evaluation is a key methodology in achieving this aim [17]. Process evaluations commonly include an account of the fidelity of intervention delivery, the ‘dose–response relationship’ between exposure to the intervention and outcomes, considerations of reach and uptake, and qualitative feedback based on participants’ experiences [18]. A combination of multiple sources of such information is desirable, but an exhaustive list is not essential to provide insight into intervention processes [19]. Evaluating the association between theoretically defined process variables and outcomes (i.e., assessing *how* interventions work) is also an important way to test the utility of specific models of behavior change and their associated intervention strategies [18,20]. Such research can lead to the refinement of intervention strategies, and of the underlying theories of health behavior change [21].

A systematic process evaluation starts with specifying the exact behavior change techniques included in a given intervention [14,15,22], and linking these to specific theoretical mediators of behavior change (e.g., a strategy of setting graded tasks might be included to promote self-efficacy) [23]. The hypothesized effects of specific techniques in influencing theoretical mediators of change, and subsequently the influence of theoretical mediators on behavioral outcomes, can then be formally tested to assess the mechanism (or multiple mechanisms) of behavior change. As such, it is no longer sufficient to report only on an intervention’s primary outcomes, it is also necessary to report its intermediary effects. The process of intervention specification can be facilitated by using standardized definitions such as those proposed in a taxonomy of behavior change techniques [24,25]. More studies that include this kind of detailed process evaluation are needed to improve our understanding of how long-term behavior change can best be achieved [26,27].

The aim of the present study was to conduct a process evaluation of a pilot trial (the Waste the Waist study) to reduce weight and cardiovascular risk through lifestyle change. Specific objectives were to test the validity of the theoretical model underpinning the intervention, and identify areas for refinement ahead of a fully powered randomized controlled trial. Full details on the development of the Waste the Waist intervention are provided elsewhere [23]. In brief, Waste the Waist was adapted from the Greater Green Triangle Diabetes Prevention Project (GGT DPP) [28], which has demonstrated the links between theoretically specified processes and the clinical outcomes of weight and waist circumference [8]. Adaptation was conducted through a systematic process of intervention mapping [29]. In line with this approach, a needs assessment was first conducted to identify determinants of behavior and behavior change for our client group, matrices of change objectives were then prepared to specify what people need to change (proximal performance objectives) in order to achieve the overarching aims of the intervention (i.e., lose weight), and matching these to theory-informed intervention components and practical strategies to achieve this. We drew on published behavior change taxonomies [24] and their supporting research [26] to populate the intervention content.

**Figure 1:**
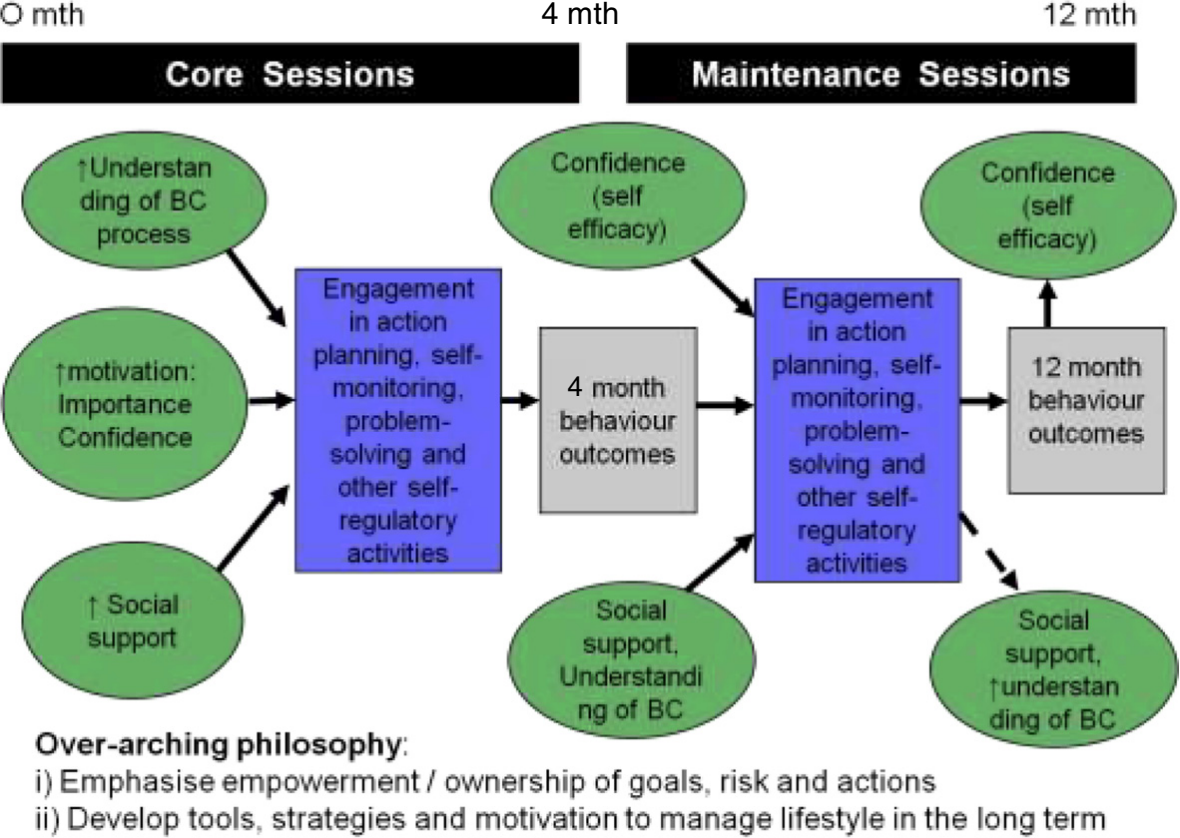
**A framework for testing hypotheses derived from the process model for lifestyle behavior change.** Notes: BC – behavior change.

The central aims of the GGT DPP and Waste the Waist interventions were to; decrease weight, reduce fat intake, reduce saturated fat intake, increase fiber consumption and increase physical activity [30]. The original GGT DPP intervention and its theoretical basis was adapted for the present UK-based cardiovascular risk group through a process of intervention mapping [23]. The theoretical model, the Process Model for Lifestyle Behavior Change (PMLBC), is a modified version of the Health Action Process Approach [HAPA; 31]. The model depicts a set of processes that result in behavior change through (a) increasing autonomous motivation (perceived importance of healthy lifestyle, self-efficacy for achieving healthy lifestyle, perceived risk and outcome expectations) and (b) promoting the formation of specific action plans (including coping plans to overcome barriers and plans for obtaining social support). Maintenance of initial changes is supported through repeated ‘self-regulatory cycles’ of feedback and reflection, involving self-monitoring, relapse prevention and reviewing/updating of goals. A framework for testing hypotheses derived from the PMLBC is presented in Figure 1.

The intervention involved a series of nine semi-structured group meetings over a nine month period designed to provide participants with the support, knowledge, skills and understanding to enable them to identify and overcome the different challenges faced in the adoption and maintenance of a healthy diet and physically active lifestyle. The data were recorded as part of a pilot randomized controlled trial conducted in south west England, and form part of the overall process evaluation alongside qualitative interviews and information on fidelity to protocol.

Starting with the premise that the intervention would be successful in bringing about changes in diet and physical activity (Hypothesis 1), the process evaluation was conducted in a hierarchical fashion reflecting three potential levels of influence on the primary outcome (weight loss); a basic physiological effect (whereby change in lifestyle behaviors leads to weight loss), an exposure (dose–response) effect, and an effect of psychological processes on lifestyle behaviors. At the physiological level, we predicted that a change in diet by reducing fat (and specifically saturated fat) and increasing fiber intake [32], and increasing physical activity [33] would result in weight loss (Hypothesis 2). To assess dose–response effects, we predicted that more regular attendance would result in greater effects (Hypothesis 3).

At the psychological level, we first expected that (within the intervention group) the quality of coach and intra-group interactions (participants’ perceptions of the support provided by coaches and satisfaction with the group setting) would moderate changes in lifestyle behaviors (Hypothesis 4). Where significant changes in behavior were observed, the utility of the PMLBC in explaining these changes was investigated. Specifically, we predicted that the intervention would lead to improvements in the following factors: increasing understanding of the process of behavior change, increasing the perceived importance of change, increasing self-efficacy and increasing social support (Hypothesis 5). We predicted that improvements in these constructs would help participants to increase their level of engagement in (or ‘enactment’ of) action planning, coping planning and self-regulation activities, as was advocated during intervention sessions (Hypothesis 6). Engagement in these planning and self-regulatory activities was predicted to at least partly mediate the relationship between psychosocial factors (motivation, social support and understanding), and changes in dietary and physical activity behaviors (Hypothesis 7). These effects were examined at four and 12 months following the start of the intervention.

In order to further inform theoretical development, we examined the potential role of affective evaluations of diet and physical activity and impulsive eating based on a dual process theory [34,35] in addition to the rational processes central to the HAPA model. Affect has been shown to be a key mediator of both physical activity and dietary behaviors [28,36,37], and is thus linked to the development of overweight and obesity [38,39]. Impulse control (the ability to resist urges to eat unhealthy food or snacks) has also been strongly associated with weight gain [40]. Therefore, in Hypothesis 8 we tested the prediction that enjoyment of diet and physical activity would be associated with the adoption of health behaviors and weight loss, and that enjoyment would have an additional independent effect on these outcomes once associations with psychological processes had been taken into account. Hypothesis 9 investigated whether the intervention was successful in improving participants’ abilities to control impulsive eating and to apply cognitive restraint when faced with temptation.

## Method

### Participants

Participants were recruited from patients registered at six General Practices in south west England. Practices identified potential participants using data from NHS Health Checks (a national cardiovascular risk screening program that started in England in 2009 [41]) and by searching computerized practice databases for risk factors. Information about the study and invitations to take part were sent to all identified patients by practice staff. We recruited people aged 40–74 with a body mass index (BMI) of 28 Kg/m^2^ - 45 Kg/m^2^*and* with high cardiovascular risk. High cardiovascular risk was defined as any combination of a) a ten-year cardiovascular risk score of 20% or more (calculated from clinical data using either the Framingham or QRISK2 algorithm), b) impaired glucose regulation defined as either a 2-hour glucose of 7.8 to 11.0 mmol/l (Impaired Glucose Tolerance) or a fasting plasma glucose of 6.1 to 6.9 mmol/l (Impaired Fasting Glycaemia), c) having hypertension, hypercholesterolemia, family history of diabetes or heart disease, history of gestational diabetes, or polycystic ovary syndrome. We excluded people with existing heart disease or type 2 diabetes, people who were pregnant or currently using weight loss drugs, people not fluent in English, people with terminal illness and anyone who, in their General Practitioner’s opinion, had other co-morbidities which would prevent engagement with the intervention.

### Intervention

The GGT DPP intervention was adapted for the local population through a systematic process of design and adaptation [28], resulting in the addition of 13 techniques and practical adjustments to reflect the needs of the patient population and local context [23]. New materials were developed for lifestyle coaches and participants to reflect the adaptations made. The intervention comprised a series of nine 2-hour long group sessions involving 8 to 12 participants, facilitated by a pair of lifestyle coaches. As social support has been demonstrated to be beneficial in facilitating weight loss [27], participants were invited to bring along a partner if they wished. Each session comprised a series of short sections to elicit and exchange ideas (e.g., about the importance of exercise, risks of excess weight, healthy eating etc.) using patient-centered counseling techniques [42]. Group activities were designed to teach key facts about diet and physical activity, in addition to the skills of action/coping planning, self-monitoring and problem-solving. Early sessions focused on the skills and information required to adopt a new behavior, and later sessions introduced discussions more relevant to the maintenance of behavior, such as dealing with stress and challenging situations, and how to maintain motivation if weight loss ‘plateaus’. Sessions also encouraged emotional self-regulation, and included a cognitive behavioral therapy technique for impulse control.

The main focus of sessions was to equip participants with a better understanding of what a healthy lifestyle is and why it is important, to encourage them towards the continued use of self-regulatory activities (goal-setting, self-monitoring of behavior and weight, reviewing progress, problem-solving and review of goals) and to help them to better understand the process of behavior change over the long term. At the start and end of each session participants were reminded of the program’s two key messages designed to encourage sustainable lifestyle change; (i) small changes can make a big difference to your weight and your health, and (ii) aim for a lifestyle that is both healthy and enjoyable (make changes that you can live with). Participants were provided with a handbook including information for reference, and were given “take away” tasks each week; these usually included implementing action plans set during session time. Details of the session content and behavior change techniques used are provided in Additional file 1.

### Procedure

Ethical approval was granted by the SW2 NHS Research Ethics Committee. Participants responding to invitation letters attended their local GP surgery for baseline data collection. They were provided with an opportunity to discuss the study further with a member of the research team, following which written consent was obtained. The researcher recorded biometric measurements and asked participants to complete self-report measures. Patients were then randomized, and participants allocated to the intervention condition directed to their nearest or most convenient group session. Sessions were held in meeting rooms in community venues close to (or based within) participating GP surgeries. Sessions initially ran weekly (Sessions 1 to 4), then fortnightly (Sessions 5 and 6), and then with longer intervals (Sessions 7, 8 and 9 were run 4, 6 and 8 months after Session 1). The six lifestyle coaches employed had a variety of backgrounds and experience including group-based counselling (n = 1), academic qualifications in nutrition or physical activity (n = 2) and fitness industry/lifestyle coaching (n = 4), and were trained by the co-authors (primarily CG, FG, AS) over 2.5 days. One of the co-authors (FG) co-delivered one series of group sessions to cover a staff shortage. Adherence to the study protocol by lifestyle coaches was promoted through; emphasis of the rationale for all intervention elements and the importance of all participants receiving the same intervention, provision of training manuals including semi-structured guides and slidesets for each session, paid preparation time ahead of each session, co-delivery by two coaches, the requirement to complete a session checklist, discussion and formative feedback given at bi-monthly supervision /de-brief meetings. Participant attendance was recorded by the lifestyle coaches.

The study protocol is published on the International Current Controlled Trials Register (ISRCTN10707899) and the study procedures were reviewed and approved by the NHS National Research Ethics Service SW Research Ethics Committee. The results are reported according to the CONSORT guidance for reporting of non-pharmacological interventions [43], the TIDierR guidelines for intervention description and replication [44] (see Additional file 2), and the theory coding scheme for good practice in intervention reporting [16].

### Measures

Measures were selected or adapted to meet the following criteria: (i) brevity (ideally 4 items or fewer), (ii) evidence of construct validity and internal reliability, and (iii) sensitivity to change demonstrated in a dietary or physical activity intervention setting. Where optimal measures could not be found, in order to maximise sensitivity to change, we constructed and piloted brief items ourselves based on concepts that were directly targeted by the intervention.

#### Understanding the process of behavior change

No existing measure of the degree to which participants understood the process of behavior change was available, so a new measure was constructed. Items were drafted and refined following piloting with 15 people (12 lay people and 3 experts), resulting in an eight-item questionnaire closely aligned to the model of change promoted in the intervention sessions (i.e., knowing how to get and stay motivated, knowing how to overcome barriers and having skills to self-regulate behavior and to manage food cravings). Responses were recorded on a 5-point Likert scale (see Additional file 3 for full questionnaire battery).

#### Perceived importance

Participants were provided with a brief definition of a healthy diet and a healthy level of physical activity, which was consistent with the intervention materials (e.g., “…eating a diet that is low in fat, low in saturated fat and that includes plenty of fruit and vegetables and plenty of starchy foods (like potatoes, pasta, rice and cereals)”.) The perceived importance of “eating a healthy diet” or “getting a healthy amount of physical activity” was then measured through two different means to facilitate a comparison of the sensitivity of a brief vs. longer measure; (1) a visual analogue scale (VAS) asking participants to rate importance from 0 (not at all important) to 10 (extremely important), and (2) an adapted version of the importance subscale of the Intrinsic Motivation Inventory [IMI; 45]. The latter scale has been demonstrated to have good reliability and validity in the context of dietary change for weight loss [46], and was adapted by asking about the level of agreement with four statements about motivations that could be important to the target group, and which were targeted by the Waste the Waist intervention; “helping to control my weight; reducing my risk of getting heart disease; contributing to my sense of well-being; could be beneficial to me.”

#### Self-efficacy

Self-efficacy for healthy eating was assessed using the 5-item version of the Weight Efficacy Life-Style Questionnaire [47]. Self-efficacy for physical activity was assessed using a 5-item scale adapted for UK vocabulary (e.g., replacing “vacation” with “holiday”) [48]. Past work has shown both measures to have good reliability and validity [47,48].

#### Social support

Social support for eating a healthy diet was measured using a 6-item measure on which participants rate how often in the last 30 days they have received different types of support or hindrance from family and friends (e.g., encouragement to eat healthy foods, complaining about their eating of healthy foods etc.), using a 5-point Likert scale [49]. Social support for physical activity was measured through a 5-item measure, using a similar format, which has been shown to have good reliability and validity in a sample of overweight adults in the USA [50].

#### Action planning

Engagement with action-planning and coping-planning for both diet and physical activity was assessed using a four-item and three-item measure respectively from the instrument developed by Sniehotta et al. [51]. To reduce participant burden, two of the five items from the original scale were omitted (‘identifying good opportunities for action’ and ‘acting in line with intentions’) as they were considered to be more distant from the central construct of coping-planning than other items.

#### Self-regulation

Engagement in self-regulation of diet and physical activity was assessed through four items for each behavior, targeting self-monitoring (two items [52]), and problem solving (two newly constructed items; i.e., *how often in the last month have I kept track of things that help me or stop me from getting enough exercise; thought about how I can overcome any problems that might stop me from getting enough exercise*).

#### Group facilitator support

Perceived support for participant autonomy provided by each of the two group facilitators was measured using four items selected from the six-item Learning Climate Questionnaire [53] that were considered the most applicable to our intervention. The LCQ has been shown to have good internal reliability and adequate construct validity in past work [54]. As correlations between scores for primary and secondary facilitators were high (r = 0.98), a single measure was computed from the mean of the two ratings for use in the analyses.

#### Group environment

Eleven items were selected from the 21-item Group Environment Questionnaire [55] to assess the quality of group experience for the individual in relation to his /her participation in the weight management group. Items were clustered into four subscales; attraction to the joint task of the group (e.g., “*this group provides me with a good opportunity to improve my lifestyle*”; 3 items), attraction to other members socially within the group (e.g., “*I enjoy my social interactions within this group”;* 5 items), perceived integration of the group towards a shared goal (e.g., “*our group is united in its belief about the benefits of healthy eating*”; 4 items), and perceived social integration (e.g., “*members of our group often socialize during sessions*”; 2 items).

#### Affective responses

Enjoyment of physical activity was assessed using an abbreviated (4-item) version of the 8-item Physical Activity Enjoyment Scale [PACES; 56]. Enjoyment of eating a healthy diet was assessed using 2 items from the Interest /Enjoyment scale of the Intrinsic Motivation Inventory [45] asking about enjoyment of “healthy foods”, and agreement with the additional statement “*I have found a diet that is both healthy and enjoyable*”.

#### Emotional self-regulation

Impulse control and the ability to manage food cravings were assessed using 10 items from the 18-item version of the Three Factor Eating Questionnaire [40]. The selected items represented the sub-scales for ‘cognitive restraint’ (all 6 items) and ‘uncontrolled eating’ (4 of 9 items).

### Behavioral outcomes

Dietary intake was assessed using the 15-item DINE food frequency questionnaire [57]. The questionnaire records the frequency over a typical week of consumption of the key food groups which account for the majority of fat and fiber intake in a UK diet. The questionnaire does not estimate energy intake, but assigns a score indicative of the amount of fat and fiber consumed relative to recommended daily averages. Although the intervention aimed to reduce both total fat and saturated fat intake, for parsimony (given that this was a pilot trial), only total fat intake was used in the analyses. The DINE measure has been shown to be sensitive to change, and valid in terms of providing results consistent with changes in blood pressure and cholesterol [58].

#### Physical activity

Was assessed using Actigraph GT3XE accelerometers, with analyses based on minutes of moderate-to-vigorous physical activity (MVPA) per registered minute of wear-time. Only participants providing four or more days of valid data (i.e., four days with over 10 hours of wear time, including one weekend day) over the data collection period (seven days) were included in the analyses. Accelerometer data were processed using Actigraph Version 6 software and a protocol successfully used in previous studies [59].

### Analysis

As the sample size of this pilot study precluded the simultaneous estimation of all proposed effects, the hypothesized relationships in Figure 1 were analyzed through a series of regression and ANOVA analyses^a^. Hypotheses 1, 5 and 9 were analyzed using 2 × 2 ANCOVA analyses, comparing change in values over time from pre- to post-intervention, between the intervention and control groups. Separate analyses were conducted for changes in outcomes from 0 to 4, and 0 to 12 months. Hypotheses 2, 3, 6 and 8 were analyzed using multiple linear regression, with change in observed outcome variables as the dependent variable; change scores were used to control for baseline values, and group allocation included as an independent variable for hypotheses 6 and 8 (hypotheses 2 and 3 only included intervention group participants). To ensure parsimony, variables were only entered into the model where significant associations between variables had been established through preliminary bivariate correlation analyses [60].

Moderation (Hypothesis 4) was assessed through hierarchical regression, entering independent variables as step 1, predicted moderating variables as step 2, and an interaction term as step 3; moderation is demonstrated if the interaction term adds significant explanatory variance to the regression model [61]. Mediation (Hypothesis 7) was explored through calculating bootstrap confidence intervals of indirect effects for hypothesized mediated relationships based on 5000 iterations [62], including all participants and controlling for group allocation. For analyses of relationships between process variables and outcomes, only reported values for cases providing data at each time-point were used to ensure that mechanisms of effect were explored in relation to actual, rather than imputed values. To provide a conservative test of efficacy in assessing Hypotheses 1 and 2 only, changes in outcomes of weight, physical activity and dietary intake were reported on an intent to treat basis computed through last observation carried forward (LOCF).

## Results

The final sample comprised 108 participants (33% female), with age ranging from 46 to 75 years (M = 65.2, SD 7.0). Forty four percent had completed their schooling by age 16, and 25% had a university degree. Mean BMI at baseline was 32.7 Kg/m^2^ (SD = 3.1), and waist circumference 103.2 cm in women (SD = 8.9) and 113.1 (SD = 8.5) in men. Fifty-eight percent of participants had a family history of heart disease, and 20% a family history of diabetes. The majority of missing data was as a result of drop-out from the trial (N = 12 failed to provide weight data (primary outcomes) at 12 months; 11%). A very small number of participants failed to complete all questionnaire items citing lack of time or difficulty. Missing data rates are higher for physical activity outcomes at some time points due to participants failing to provide minimally acceptable data.

Most variables approximated a normal distribution, and were positively skewed. However, perceived importance demonstrated a ceiling effect for both diet and physical activity (M > 6.20 on a 1–7 point scale for both measures). Although scores were also approaching ceiling levels using the VAS (M = 8.0 for physical activity and 8.6 for diet on a 1–10 scale), there was more scope using this measure to detect an increase. Consequently, the VAS measure was used in the analyses. All measures demonstrated acceptable internal consistency (Cronbach’s α values range from 0.7 to 0.9; see Additional file 4: Table S1).

### Main analyses

#### Did the intervention result in weight loss and behavior change?

Hypothesis 1: At four months the intervention resulted in a significant reduction in weight (M difference −2.0 Kg. 95% CI: −3.3 to −0.7, p = 0.001), a significant increase in fiber consumption (M difference =5.7 g/day, F(1,106) = 15.1, p < 0.001) and a non-significant reduction in fat consumption (M difference = −2.3 g/day, F(1,106) = 2.3, p = 0.13) (Table 1). At 12 months the increase in fiber intake remained significant (F(1,105) = 9.2, p < 0.005). The difference in weight loss fell short of significance (intervention group −3.7 kg, SD = 5.2 vs control group −1.9 kg, SD = 6.7; F(1,108) = 2.3, p = 0.13), although when adjusted for co-interventions (i.e., medical treatment) and incident morbidities that might affect weight, the difference was significant (M Difference 2.9 kg, 95% CI: −5.1 to −0.6, p = 0.014). There was no significant difference in objectively measured physical activity at four or 12 months post intervention.

**Table 1:** **Changes in outcomes at four and 12 months post intervention**

	**Control group mean** (**SD)**			**Intervention group mean (SD)**			**Effect size (** ***d*** **)** ^**a**^
	**Baseline**	**4 months**	**12 months**	**Baseline**	**4 months**	**12 months**	**4 months**
Weight (kg)	N = 52 97.57 (12.84)	N = 48 96.65 (12.55)	N = 47 95.67 (12.39)	N = 54 96.63 (13.96)	N = 45 93.78 (13.76)**	N = 43 92.98 (14.10)	−0.67
Diet	N = 52	N = 48	N = 47	N = 54	N = 45	N = 43	
Fat intake	32.23 (10.91)	27.57 (10.25)	28.04 (9.19)	29.98 (9.13)	24.22 (8.58)	24.98 (8.31)	−0.21
Fiber intake	36.98 (10.00)	34.40 (9.62)	34.30 (9.61)	36.72 (11.60)	39.85 (10.64)***	39.67 (11.29)**	0.70
Physical activity	N = 53	N = 44	N = 43	N = 53	N = 42	N = 42	
MVPA^b^	24.56 (17.75)	28.00 (20.12)	29.19 (23.00)	24.93 (23.51)	31.14 (21.18)	26.25 (21.09)	-.11
Activity counts^b^	270.52 (106.56)	286.20 (123.01)	287.68 (119.26)	240.60 (118.44)	266.32 (130.41)	250.85 (118.59)	-.09
Perceived importance	N = 48	N = 47	N = 45	N = 52	N = 47	N = 44	
Diet (scale 1–10)	8.49 (1.29)	8.50 (1.43)	8.23 (1.97)	8.77 (1.40)	9.26 (.92)	9.14 (1.17)	.29
PA (scale 1–10)	7.74 (1.64)	7.67 (1.95)	7.42 (2.17)	8.19 (1.98)	8.61 (1.57)*	8.30 (1.71)	.67
Self-Efficacy	N = 48	N = 47	N = 45	N = 52	N = 47	N = 44	
Diet (scale 1–8)	4.42 (1.48)	4.41 (1.77)	4.84 (1.53)	4.43 (1.64)	5.91 (1.30)***	5.89 (1.41)***	.98
PA (scale 1–8)	2.76 (.93)	2.74 (.84)	2.78 (.99)	2.93 (.89)	3.28 (.83)**	3.14 (.89)	.42
Social Support	N = 48	N = 47	N = 45	N = 52	N = 47	N = 44	
Diet (scale 1–5)	3.23 (.86)	3.20 (1.03)	3.25 (.87)	3.17 (.80)	3.39 (.91)	3.30 (.97)	.39
PA (scale 1–5)	2.29 (1.07)	2.23 (1.15)	2.09 (.99)	2.14 (1.02)	2.57 (1.04)***	2.29 (.94)*	.89
Understanding (scale 1–5)	N = 46	N = 47	N = 45	N = 52	N = 47	N = 44	
	3.21 (.65)	3.41 (.54)	3.54 (61)	3.24 (.65)	4.07 (.51)***	4.11 (.50)***	.81
Action planning	N = 46	N = 47	N = 45	N = 47	N = 46	N = 44	
Diet	2.76 (.75)	2.96 (1.20)	3.34 (1.22)	2.79 (.76)	3.54 (.83)**	3.33 (.89)	.53
	N = 47	N = 46	N = 45	N = 52	N = 46	N = 44	
PA	2.41 (.74)	2.80 (1.13)	3.11 (1.39)	2.52 (.84)	3.17 (.90)	3.11 (1.10)	.33
Coping planning	N = 46	N = 47	N = 45	N = 50	N = 46	N = 44	
Diet	2.38 (.69)	2.72 (1.30)	3.13 (1.39)	2.40 (.76)	3.25 (.93)*	3.15 (1.02)	.48
	N = 46	N = 46	N = 45	N = 52	N = 46	N = 44	
PA	2.13 (.67)	2.60 (1.22)	2.90 (1.43)	2.18 (.83)	2.99 (.93)	2.94 (1.05)	.38
Self-monitoring	N = 47	N = 47	N = 45	N = 52	N = 47	N = 44	
Diet	2.60 (.60)	2.59 (.60)	2.61 (.65)	2.52 (.48)	2.96 (.50)**	2.92 (.45)**	1.03
PA	2.32 (.56)	2.37 (.51)	2.38 (.65)	2.36 (.57)	3.01 (.61)*	2.69 (.59)*	1.00
Enjoyment	N = 46	N = 47	N = 45	N = 52	N = 46	N = 44	
Diet	4.72 (1.34)	4.76 (1.48)	4.86 (1.25)	4.83 (1.30)	5.54 (1.32)**	5.63 (1.21)**	.54
PA	3.67 (1.38)	3.63 (1.23)	3.44 (1.40)	4.00 (1.44)	3.47 (1.54)	3.61 (1.82)	-.24
Impulse control	N = 47	N = 47	N = 45	N = 52	N = 46	N = 44	
Cognitive restraint	2.35 (.52)	2.32 (.75)	2.31 (.59)	2.24 (.52)	2.68 (.64)**	2.57 (.58)**	.75
Uncontrolled eating	1.99 (.70)	1.94 (.67)	1.96 (.67)	1.99 (.68)	1.82 (.61)	1.76 (.57)*	-.17

Notes: PA – physical activity; *p < .05, **p < .01, ***p < .001; N varies due to lack of valid wear-time for PA, or non-completion of full set of measures due to participant time restraints/preference; ^a^change in intervention group from baseline, relative to change in control group; ^b^Values from log transformations were used in analyses, but raw data presented here for ease of interpretation. Only participants reporting ≥ 4 days complete accelerometry data at each time point were included. All other missing data reflects drop-out from the trial (N = 12), and refusal to complete all measures due to lack of time/interest.

Hypothesis 2: In the regression model, changes in diet (fat and fiber) were significantly predictive of weight loss at four (R^2^ = 0.2, p < 0.001) and 12 months (R^2^ = 0.1, p < 0.01), supporting Hypothesis 2. Both fat and fiber intake were independently predictive of weight loss at each time point (for fat intake β = 0.3, fiber β = −0.3).

#### Did exposure to the intervention relate to outcomes?

Hypothesis 3: Participants attended a median of seven of the nine available meetings, with 70% attending at least five sessions. Attendance was not significantly predictive of a reduction in fat (R^2^ = 0.09, p = 0.05) or an increase in fiber intake (R^2^ = 0.03, p = 0.28), but did predict greater weight loss at 4 months (R^2^ = 0.2, p < 0.005). There was no significant association between attendance and any outcome at 12 months. However, there appeared to be a substantial threshold effect; participants who attended five or more sessions lost significantly more weight at four (Mean diff = 3.7 Kg, 95% CI: 2.0 to 5.5, p < 0.001) and 12 months (M difference = 4.1 Kg, 95% CI: 1.2 to 7.1, p < 0.01), and reported greater reductions in fat intake at four months (M difference = 7.6 g/day, 95% CI: 0.7 to 14.6, p < 0.05).

Hypothesis 4: In terms of the quality of intervention experience, there was little variation and a ceiling effect for perceived autonomy support (M = 6.2, SD = 0.98; scale range 1–7). The associations between group experience factors and behavioral outcomes were relatively weak (Table 2), with significant associations reported only between attraction to the group task and self-monitoring (r = 0.4, p < 0.05) and fat intake (r = −0.3, p < 0.05). The associations between self-monitoring and task integration (r = 0.3, p = 0.06) and group social attraction (r = 0.3, p = 0.06) neared significance. No moderator effects were found, as all regression models were non-significant.

**Table 2:** **Correlations between group environment and dietary behavioural outcomes at four months**

	**N**	**Range**	**Mean (SD)**	**Action planning**	**Coping planning**	**Self-monitoring**	**Fat intake**	**Fiber intake**	**Weight loss**
Attendance	54	0-9	5.96 (2.79)	0.04	−0.10	0.05	−0.39*	0.22	−0.44*
Group cohesion	43	1-5	3.54 (0.61)	0.08	0.17	0.28	−0.25	−0.17	−0.19
Autonomy support^a^	43	1-7	6.20 (0.98)	0.12	0.20	0.25	−0.03	0.11	0.02

Notes: ^a^ = combined measure for both group facilitators; *p < .01.

#### Did the intervention result in significant changes in the proposed processes of behavior change?

Hypothesis 5: Patients’ understanding of the behavior change process and self-efficacy for improving their diet significantly increased at both four and 12 months (Table 1). Changes in the perceived importance of a healthy diet and social support were not significant, but effect sizes suggested a numerical trend towards an advantage over the control group at four months (M difference importance = 0.1, p = 0.3, d = 0.3; M difference social support = 0.3, p = 0.08, d = 0.4). Although the intervention was not successful in changing physical activity behavior there were significant improvements with moderate to large effect sizes in participants’ self-efficacy (d = 0.4), social support (d = 0.9) and perceived importance for physical activity (d = 0.7) (Table 1). Self-efficacy towards healthy eating (M difference = 1.1, p < 0.001), understanding the process of change (M difference = 0.5, p < 0.001), and social support for physical activity (M difference = 0.4, p < 0.05) remained significantly above baseline at 12 months. Thus, in relation to both diet and physical activity, Hypothesis 5 was largely supported over the 4-month behavioral adoption phase, and partially supported for the maintenance phase.

Hypothesis 6: Engagement in action planning, coping planning and self-monitoring in relation to diet and physical activity increased significantly more in the intervention than control group from baseline to four months (Table 1). At 12 months, only self-monitoring (for both behaviors) and coping-planning in relation to diet remained higher in the intervention group. At four months, greater engagement in dietary action planning (R^2^ = 0.1, p < 0.05), coping planning (R^2^ = 0.2, p < 0.01) and self-monitoring (R^2^ = 0.3, p < 0.001) were significantly predicted by a regression model including all four motivation-related process variables, controlling for group allocation. At 12-months, only self-monitoring was significantly predicted by the model (R^2^ = 0.4, p < 0.001); greater self-monitoring was predicted by improvements in motivational variables in the intervention group only.

For physical activity, only action planning (R^2^ = 0.2, p < 0.01) and self-monitoring (R^2^ = 0.4, p < 0.001) were significantly predicted by improvements in motivational process variables at four months (coping planning R^2^ = 0.1, p = 0.16). However, all three self-regulatory activities were significantly predicted by change in motivational processes at 12 months (action planning R^2^ = 0.3, p = 0.001; coping planning R^2^ = 0.2, p < 0.01; self-monitoring (R^2^ = 0.3, p < 0.001). Group allocation was only predictive of self-monitoring at 4-months; improvements in motivational constructs were only predictive of increased self-monitoring in the intervention group. Full correlation tables are available as Additional file 4: Table S2.

#### Did enactment of self-regulatory activities mediate behavior change?

Hypothesis 7: At four months, self-regulatory activities, and self-monitoring in particular, significantly mediated the relationship between understanding the behavior change process and fat intake (95% CI for Total effect = −2.9 to −0.2; 95% CI; indirect effect for self-monitoring −2.4 to −0.1); a greater improvement in understanding was associated with increased self-monitoring (R^2^ = 0.2, p < 0.01), which was in turn associated with a greater reduction in fat intake (R^2^ = −4.6, p = 0.05). Self-regulatory activities did not mediate any other predicted influences on fat intake nor the relationships between psychosocial variables and fiber intake, physical activity (MVPA), or weight loss itself. At 12 months, there were no significant mediation effects in relation to diet, but self-monitoring of physical activity significantly mediated the relationship of weight loss with both self-efficacy (CI indirect effect = 0.004 to 1.98) and understanding (CI indirect effect = 0.07 to 2.36); i.e., better understanding and self-efficacy predicted greater engagement in self-monitoring of physical activity, which in turn predicted a greater weight loss. No other mediation effects were found, and thus, only limited support was found for Hypothesis 7.

#### Did the intervention influence affective outcomes?

Hypothesis 8: The intervention did not result in a significant change in the enjoyment of physical activity, but did increase enjoyment of diet (Table 1). Although increased enjoyment was associated with weight loss at both four (r = −0.2, p < 0.05) and 12 months (r = −0.2, p < 0.05), it provided no additional independent explanatory effect on outcomes when controlling for other motivational variables (4 month R^2^ = −0.12, p = 0.8; 12 month R^2^ = 0.9, p = 0.3). Similarly, change in enjoyment did not predict any additional variation in fat (R^2^ = 0.7, p = 0.4) or fiber (R^2^ = 0.2, p < 0.05; β for enjoyment = −0.1, p = 0.4) intake in addition to motivational process variables.

Hypothesis 9: Compared with controls, the intervention resulted in a significant increase in cognitive restraint at both four (M difference = 0.4, F(1,85) = 12.3, p < .0.05) and 12 months (M difference = 0.4, F(1,83) = 19.9, p < 0.001). There was no improvement in uncontrolled eating at four months (M difference = −0.1, F(1,85) = 0.4, p = 0.4), but a significant improvement by 12 months (M difference = −0.2, F(1,83) = 50, p < 0.05 ). Changes in uncontrolled eating (controlling for group allocation and other motivational variables) significantly predicted weight loss at four months (R^2^ = 0.2, p < 0.05; β = 0.2 p < 0.05). A significant effect on weight loss was only observed in the intervention group (predicted by reduced uncontrolled eating, and increased self-efficacy). No additional explanatory power in predicting dietary or weight loss outcomes was found for the effects of cognitive restraint.

## Discussion

This process evaluation provides insight into the key processes involved in bringing about weight loss and behavior changes in a pilot study of a group-based weight loss intervention [23]. The findings provide an initial exploration of the Process Model for Lifestyle Behavior Change (PMLBC), and insight into where the proposed mechanisms broke down in failing to promote positive change in physical activity. In accordance with the secondary aims of the study to report on the sensitivity and reliability of the measures included, the findings also support the use of a new measure of ‘understanding the process of behavior change’, which was found to be a significant predictor of engagement in self-regulatory activities, and a mediator of the effect of self-monitoring on fat intake at four months, and weight at 12 months.

The findings provide some initial support for the PMLBC for bringing about changes in diet, in that the hypothesized relationships between motivational, self-regulatory and behavioral outcomes were largely as predicted, and were associated with weight loss. Furthermore, the findings show that the intervention was successful in generating changes in most of the processes targeted by the PMLBC. Over the first four months, the intervention successfully increased patients’ understanding of the process of behavior change and self-efficacy for eating a healthy diet, with moderate to large effect sizes. Although the perceived importance of eating a healthy diet was not significantly influenced by the intervention itself, participants’ baseline ratings for this construct were already high. This may reflect the fact that patients had recently been informed by their family doctor that they were at high cardiovascular risk. Even with this ceiling effect, a change of a meaningful effect size in the predicted direction was reported (d = 0.3). Similarly, although social support was not significantly improved by the intervention, there was a meaningful size of effect that was close to significance in the expected direction (d = 0.4). Improvements in all process variables except for perceived importance were associated with greater engagement with one or more self-regulatory activities. While associations between these self-regulatory activities and changes in dietary behaviors were less strong, self-monitoring appeared to mediate (a) the process by which increased self-efficacy resulted in weight loss, and (b) the relationship between greater understanding of the weight loss processes and fat intake. As these analyses are associative rather than inferential no conclusion can be made regarding causality, or whether these relationships are unidirectional or bi-directional. Future research exploring the role of action and coping planning in weight loss and dietary behavior change, and investigating the direction of effects would therefore be valuable.

Participants who had a higher level of social support reported better engagement in self-regulatory activities, particularly over the short-term. This suggests that social support is important for dietary behavior change and should be retained in the PMLBC model, but more effective strategies to enhance social support need to be developed. Social support was a key focus of all group sessions through; encouraging participants to identify who they can call on for support in their existing networks, promoting an awareness of negative social influences (i.e., social undermining) for the purposes of coping planning, and encouraging participants to set action plans for establishing social support. Feedback from lifestyle coaches indicated that while participants acknowledged the importance of positive social support to their weight loss attempts, they were reluctant to make plans to actively enlist social support from their friends and families. In retrospect, using a measure of adherence to social support planning would have provided useful quantitative evidence to support these anecdotal reports. Our findings are consistent with other research which confirms the importance of engaging social support to promote weight loss [26], but that the strategies commonly used within complex interventions are not always successful (e.g., [63]). Qualitative work to explore perceived barriers to implementing social support plans would be useful.

Prior research suggests that the adoption and maintenance phases of changing lifestyle behaviors may have different determinants, and therefore require different approaches (e.g., [64-67]). In our study there was some indication of a change in the relationship between self-regulatory behaviors and study outcomes (specifically, weight and fat intake) over time, but this was only to a limited degree. Self-monitoring of dietary intake was associated with weight loss at four months but not at 12 (although it was still significantly associated with fat intake), whereas coping planning around diet was significantly associated with weight loss at 12 months but not at four months. This is consistent with past work that reports a delayed (i.e., longer-term) effect of coping planning on physical activity behavior [68]. Coping planning is a key component of relapse prevention interventions, and considered important to sustaining weight loss by avoiding small setbacks leading to reversal to former habits [50]. Further studies involving larger participant numbers would be required to provide a more robust test of the change in associations over phases of behavior change.

Although the intervention did not increase moderate to vigorous physical activity, the process evaluation enabled us to explore where the predicted mechanisms of effect broke down. At four months, the intervention significantly increased social support, self-efficacy and perceived importance in relation to MVPA. These changes were associated with increased action and coping planning, and self-monitoring. Thus, the intervention was successful in bringing about largely equivalent changes in process variables to those brought about in relation to diet, but in the case of physical activity these were not sufficient to bring about changes in behavior. One reason for this may be that the changes in process variables were not of a sufficient magnitude to support behavior change, at least in terms of the impact on MVPA. Notably, the effect size for change in self-efficacy for MVPA was much smaller than that for eating a healthy diet (d = 0.97 for diet vs. d = .42 for physical activity). However, a recent systematic review suggests that changes of much smaller effect size (mean effect size d = 0.16; 95% confidence interval: 0.08-0.24) have been previously associated with changes in physical activity [67]. In the same review, intervention components common to the more successful studies included providing (self-initiated) rewards for effort or progress, and providing instruction which were not core components of Waste the Waist. Other reviews suggest that mechanisms other than self-efficacy may be more important for increasing the physical activity of obese individuals [69]; the behavior change techniques most strongly associated with success were ‘teach to use prompts/cues’, ‘prompt practice’ and ‘prompt rewards contingent on effort or progress towards behavior’. Further, in older people self-regulation techniques may actually be counter-productive to change (only 2 behavior change techniques were associated with success: “barrier identification/problem solving” and “model/demonstrate the behavior”) [70]. This implies that providing instruction (i.e., in the form of practical exercise sessions) to demonstrate the type and intensity of activity required and re-assure people that this is safe could represent a useful addition to the Waste the Waist intervention.

We found no significant relationship between study outcomes and participants’ evaluation of either group cohesion or autonomy support from the coach. Our measures did exhibit ceiling effects, so may not have been sufficiently sensitive to identify the hypothesized associations. It is also possible that the group environment and relationships are important only up to a threshold level (as was found for the case of attendance). Given that perceptions of the environment were largely positive in the present study, we did not have the variation in data to fully explore this hypothesis.

Our exploration of the association between affective responses and study outcomes provides feedback on the potential benefits of extending the model to encompass these additional factors. Although participants’ enjoyment of healthy eating and impulse control abilities improved as a result of the intervention, these processes did not appear to contribute independent effects towards study outcomes. Cognitive restraint did add explanatory value, and as in other studies (e.g., [71]), was found to be a significant predictor of the maintenance of weight loss outcomes. Further exploration of these factors may be useful.

### Implications for refinement of the intervention

The findings suggest several ways in which we can improve our intervention. These include; refining our strategies for engaging social support and overcoming negative social influences, refining our strategies for increasing physical activity, particularly with a view to translating increased efficacy into action (perhaps by facilitating practice in the sessions), encouraging self-initiated rewards for success, and providing prompts (e.g. text or email reminders). If we can enhance the intervention such that it also promotes positive changes in physical activity in addition to diet, and to have a positive impact on social support, it is likely that stronger effects on weight loss will be achieved.

### Strengths and limitations

A key strength of the current study is the systematic way in which the model of change was specified in line with best practice in intervention design [14,16,19]. Depending on their focus, process evaluations have the potential to enhance the impact of intervention research by increasing our understanding of the interactions between the factors influencing behavior at multiple levels (e.g., individual, interpersonal, contextual), of dose and implementation effects on outcomes, and to contribute towards theory development [19]. The Waste the Waist intervention built on past work through the inclusion of mechanisms that have previously shown promise in interventions designed to reduce cardiovascular risk through lifestyle change [5,28,72]. This process evaluation thus provides feedback on the performance of specific behavior change techniques in influencing the hypothesized mediators of change, in addition to evaluating the model itself (the PMLBC). This process evaluation also provides new information on the sensitivity, reliability and validity of a range of short measures (including some new measures) that have a low response burden and are appropriate for use as process variables in future trials. In particular, the finding that reduced-length scales such as the VAS for measuring perceived importance seem to have good reliability, construct validity and sensitivity to change provides one means of reducing participant burden in similar process evaluations. This is the first study to incorporate a process variable measuring participants’ understanding of the *process* of behavior change. This variable helped to explain changes in self-regulatory and behavioral outcomes at both four and 12 months. This intervention technique may therefore be a useful component in weight loss interventions and should be added to taxonomies of behavior change techniques.

The main limitations to this study were relatively low sample size (as above) which precluded the analysis of simultaneous direct and indirect paths (e.g., through structural equation or growth modelling), and likely measurement error due to the use of self-report for most process variables. This was a pilot trial, so there was not necessarily sufficient power for all process analyses. As such, the findings provide preliminary rather than conclusive results on the significance of the proposed mechanisms of effect. Further, although the data are broadly supportive of the hypotheses generated from the PMLBC, the majority of the analyses are associative rather than inferential. Hence, we cannot make inferences about the causal nature of the relationships between process variables and behaviour change, or whether these relationships are unidirectional or bi-directional. On the whole, the process measures seemed to perform well (Additional file 4: Table S1 provides the psychometric properties), exhibiting good internal reliability and sensitivity to the intervention. However, ceiling effects in perceived importance measures and the group delivery measures may have led to underestimation of associations with these factors.

## Conclusions

The results of this pilot study provide insight into the mechanisms responsible for bringing about positive changes in behavior and weight in the Waste the Waist intervention. The proposed PMLBC model was largely supported for the promotion of dietary change. However the model or the strategies used to bring about change in its constituent components need to be adapted for promoting physical activity, or alternative models tested. The sustained effects on process variables over a 12 month period indicate that the intervention brought about lasting effects on participants’ motivation and cognitions, and that these were associated with sustained effects on dietary intake and weight. Further work on a larger sample is warranted to explore the model in more detail. Refinements to address aspects of the model that were not significantly influenced by the intervention (e.g., engagement in action and coping planning for physical activity, the promotion of social support for eating a healthy diet), need to be applied to the Waste the Waist intervention ahead of testing the model in a full-scale intervention trial.

The present study also provides initial support for the utility of a new measure assessing participants’ understanding of the process of behavior change. This construct explained a significant amount of variance in engagement in self-regulatory behaviors, diet and weight in addition to that explained by standard motivational constructs. Further research to explore the role of ‘teaching participants how behavior change works’ in bringing about sustained behavior change would be informative.

## Endnote

^a^We did not control for potential clustering effects of patients by GP practice, as the intra-cluster correlation coefficient (ICC) for clustering of weight loss (0–12 months) by GP practice was 0.000 (95% CI 0.000, 0.084) for the whole sample. The coefficient was similar when examined within each group.

## Additional files

Additional file 1:
**The Waste the Waist Intervention.**
Additional file 2:
**Checklist of Items for Reporting Trials of Nonpharmacologic Treatments*.**
Additional file 3:
**Selection of Measures for Waste the Waist Evaluation.**
Additional file 4: Tables S1 and S2. Distribution and psychometric properties of study variables at baseline.
