# Supplementary material for: Processes of behavior change and weight loss in a theory-based weight loss intervention program: a test of the process model for lifestyle behavior change
Source: Int J Behav Nutr Phys Act. 2015 Jan 16;12:2. doi: 10.1186/s12966-014-0160-6 (PMC4304200; doi:10.1186/s12966-014-0160-6)
Supplement: Additional file 1: — The Waste the Waist Intervention. [file 12966_2014_160_MOESM1_ESM.docx]

**The Waste the Waist Intervention**

The Waste the Waist intervention was based on the Australian “Greater Green Triangle” (GGT) Programme.[1] This programme was selected as it a) produces more than 2Kg mean weight loss in people with elevated cardiovascular risk b) modifies other cardiovascular risk factors (plasma glucose, cholesterol, triglycerides, blood pressure) c) can be successfully delivered on a large scale[2] d) is within a cost threshold set by our primary care and public health stakeholders (less than £400 per person) e) includes, or can be modified to include, additional intervention components recommended by relevant systematic reviews[3-5] and advice from our service user advisory group and f) has a clearly defined theoretical basis.[6]

*Aims:* The intervention aimed to encourage weight loss by increasing physical activity, reducing intake of total and saturated fat, increasing fibre intake and other dietary changes (such as reducing portion sizes). Targets were set by participants, but the health benefits of 5% weight loss and of 150 mins per week of moderate activity were presented and suggested as minimum long-term targets for health gain.

*Theoretical model:* We extended the intervention and its theoretical model (the Health Action Process Approach[7]) by adding a number of behaviour change strategies to include a greater emphasis on social support, self-monitoring and relapse management and the use of coping plans.[8] Following a suggestion from our service-user group, we also added a novel element - explaining the process of behaviour change to participants. The process model for the Waste the Waist intervention is illustrated in Fig.1. The intervention processes involved a) increasing motivation (defined as perceived importance of healthy lifestyle, self-efficacy for achieving healthy lifestyle, perceived risk and outcome expectations); b) making a specific action plan (including plans for social support and for overcoming barriers (coping plans)) and c) supporting maintenance through repeated ‘self-regulatory cycles’ of feedback /reflection, use of self-monitoring and relapse prevention techniques and revision of action plans. Detail of the behaviour change techniques included in the intervention is presented in Appendix 1 (p7-10)

***Content:*** An overview of the content and timings for each session is provided in Appendix 2 below. The behaviour change techniques used to promote changes in the targeted processes are illustrated in Fig.2. These included motivational interviewing, self-assessment of risk factors (diet and physical activity levels), identifying and engaging sources of social support /addressing social influences, SMART-R goal setting (Specific, Measurable, Achievable, Relevant, Time-related, Repeatable), making coping plans and social support plans, self-monitoring of outcomes (using self-weighing and the participants’ choice of pedometers or physical activity diaries), frequent reviews of progress, problem-solving and goal review. We had a strong emphasis on empowering participants to develop and practice skills for lifestyle behaviour change. This included discussing how the process of behaviour change works and why they were being asked to engage in the different activities (such as action planning and self-monitoring). Thus, we made the model we were using (Fig.1) explicit from the outset.

To promote sustainability of weight loss we advised participants to make a series of small, achievable changes, rather than dramatic, unsustainable changes. We encouraged participants to prioritise ideas for change that would not detract from their enjoyment of food (for dietary changes) or that would be enjoyable or easy to build into a routine (for physical activity).[9] Key messages that were repeated throughout the programme were “Make changes you can live with”, “Small changes make a big difference” and “Aim for a lifestyle that is both healthy and enjoyable”. We also included one technique based on cognitive behavioural therapy (using a mental ‘STOP’ sign and some pre-conceived strategies to address ‘in the moment’ food cravings).

Throughout the intervention we provided and discussed information about

1. Healthy eating - we used the Public Health England “Eat Well Plate”[10] as a guide and intervention tool to prompt ideas for reducing calorie intake by replacing fat (especially saturated fat) with fruit, vegetables and high fibre carbohydrates, reducing intake of high fat /high sugar snacks and reducing overall calorie intake.
2. Physical activity – including how to gauge intensity, especially in relation to walking pace and discussing ideas and opportunities for increasing physical activity. A set of home-based resistance exercises (as per government guidance to engage in muscle-strengthening exercise involving all muscle groups on at least two days of the week[11]) was introduced and discussed. However, no demonstration or structured exercise classes were provided.

Targets were set by participants, but the health benefits of 5% weight loss and of 150 minutes of moderate intensity physical activity were presented and suggested as minimum long-term targets for health gain.

*Training and delivery:* The style of delivery was considered to be important and we trained our lifestyle coaches to use person-centred counselling techniques derived from motivational interviewing (open questioning, affirmation, reflective listening, summaries, use of the elicit-provide-elicit (e-p-e) technique for information exchange)[12, 13] to deliver all of the intervention content. We recruited seven lifestyle coaches from the local community with varied backgrounds and experience, including group-based counselling (1), academic qualifications in nutrition or physical activity (2) and fitness industry /lifestyle coaching (4). A 2.5 day training course was developed and delivered by the co-authors (primarily CG, FG, AS).

We used session plans and slide-sets for each session to structure the intervention and used icons (pause buttons, flipchart symbols) to remind the lifestyle coaches to stop the slideshow and engage in discussion and other interactive activities specified in the session plan. Written manuals and materials were produced to support (and document) both the intervention and its training course.

The Waste the Waist intervention was delivered in local community venues (e.g. community halls, meeting rooms in GP practices after hours). The intervention consisted of four 120-minute group based sessions in the first month to support initial behaviour change, then five 90-minute maintenance support sessions at 1.5, 2, 4, 6 and 9 months after the first session. The total contact time was therefore 13.5 hours spread over 9 months. Groups consisted of 8-12 participants, facilitated by two lifestyle coaches.

**Fig.S1: The Process Model of Lifestyle Behaviour Change.[14-16]**


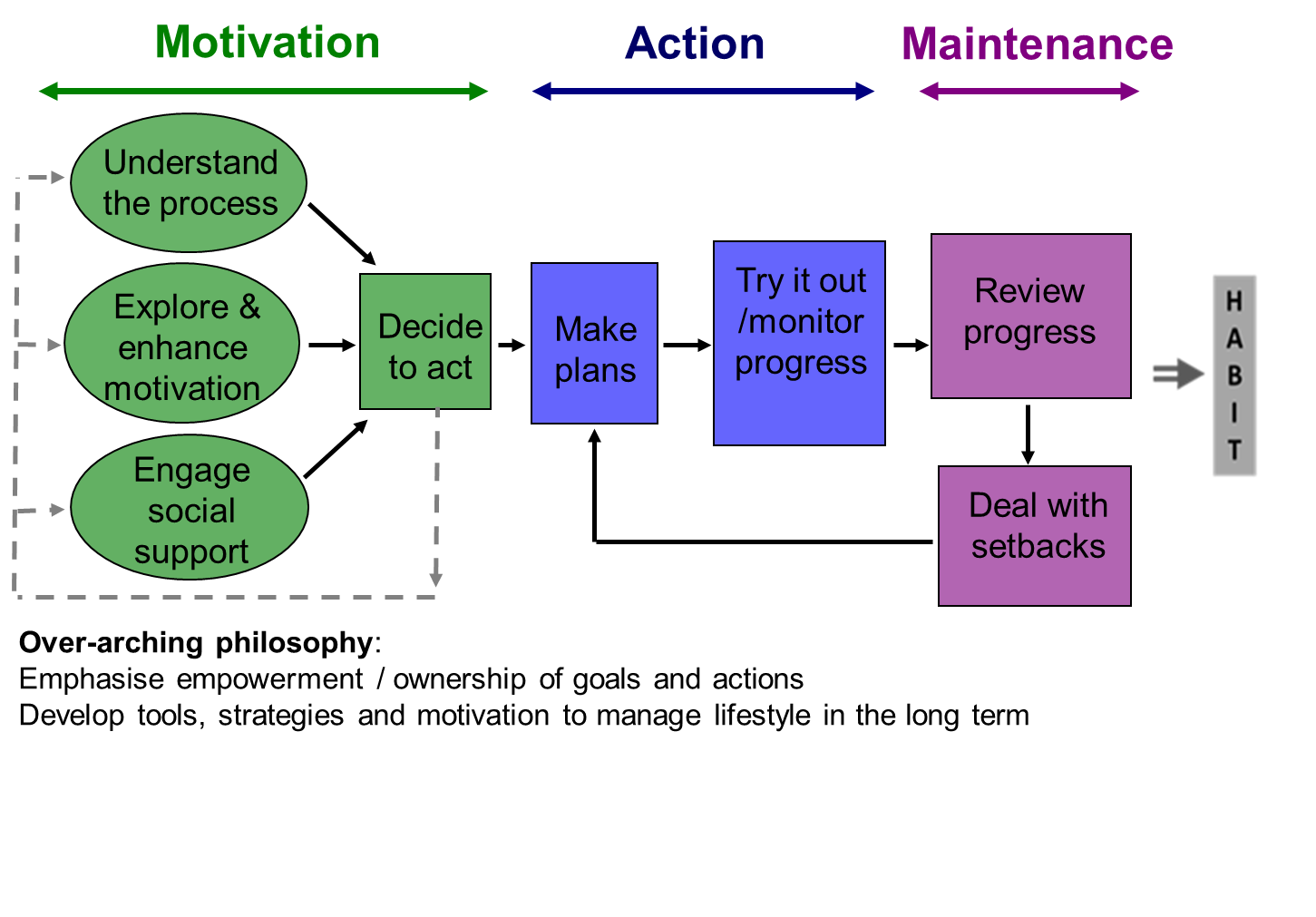


**Fig.S2: Behaviour change techniques used in Waste the Waist**


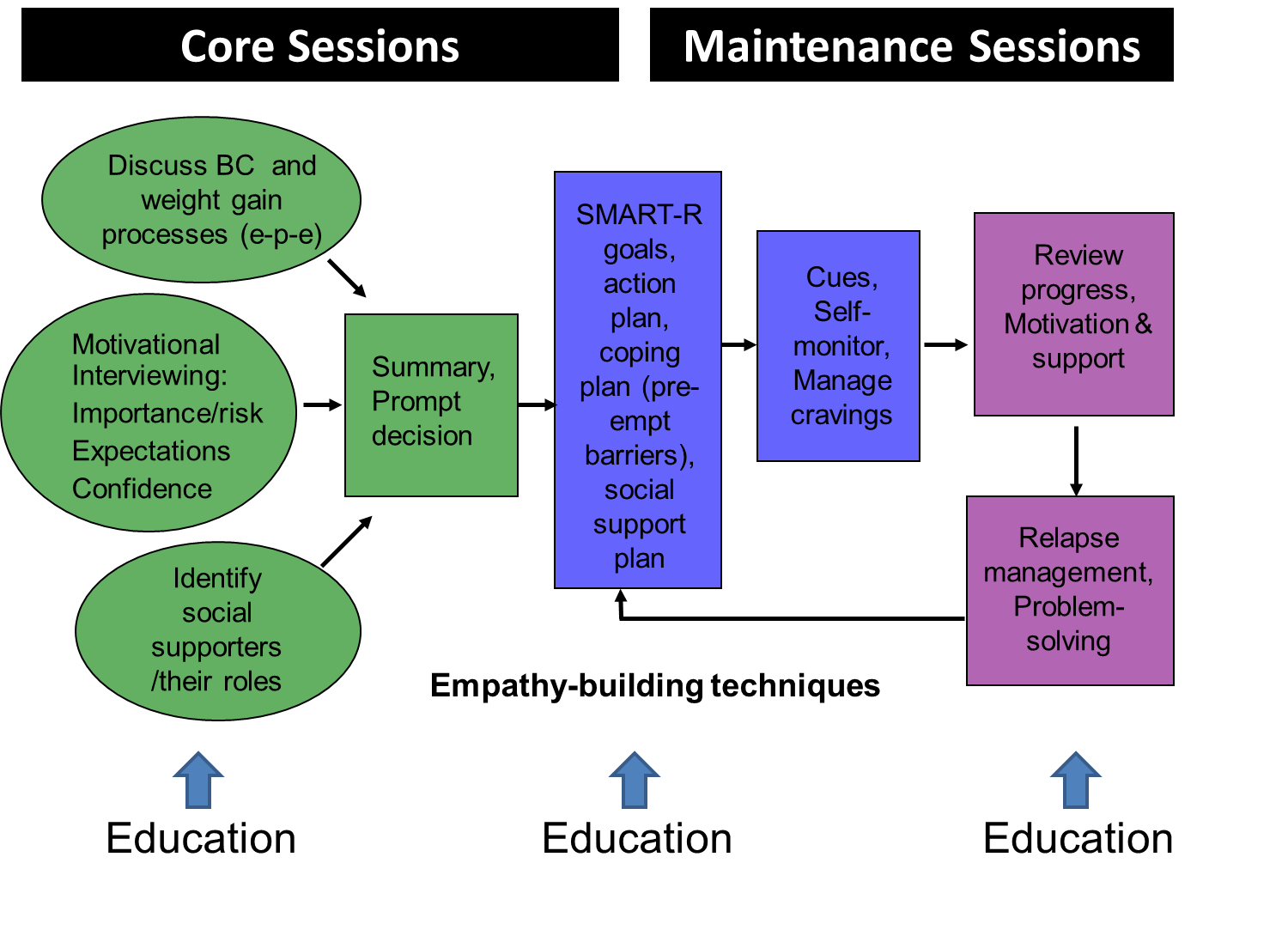


1. Laatikainen T, Dunbar J, Chapman A, Kilkkinen A, Vartiainen E, Heistaro S, Philpot B, Absetz P, Bunker S, O'Neil A, et al: **Prevention of Type 2 Diabetes by lifestyle intervention in an Australian primary health care setting: Greater Green Triangle (GGT) Diabetes Prevention Project.** *BMC Public Health* 2007, **7:**249.

2. Dunbar JA, Jayawardena A, Johnson G, Roger K, Timoshanko A, Versace VL, Shill J, Philpot B, Vartiainen E, Laatikainen T, et al: **Scaling up diabetes prevention in Victoria, Australia: policy development, implementation and evaluation.** *Diabetes Care* 2013.

3. Greaves CJ, Sheppard KE, Abraham C, Hardeman W, Schwarz P, Roden M, The IMAGE Study Group: **Systematic review of reviews of intervention components associated with increased effectiveness in dietary and physical activity interventions.** *BMC Public Health* 2011, **11:**1-12.

4. Dombrowski SU, Sniehotta FF, Avenell A, Johnston M, MacLennan G, Araújo-Soares V: **Identifying active ingredients in complex behavioural interventions for obese adults with obesity-related co-morbidities or additional risk factors for co-morbidities: a systematic review.** *Health Psychology Review* 2012, **6:**7-32.

5. Artinian NT, Fletcher GF, Mozaffarian D, Kris-Etherton P, Van Horn L, Lichtenstein AH, Kumanyika S, Kraus WE, Fleg JL, Redeker NS, et al: **Interventions to promote physical activity and dietary lifestyle changes for cardiovascular risk factor reduction in adults: A scientific statement from the American Heart Association.** *Circulation* 2010, **122:**406-441.

6. Uutela A, Absetz P, Nissinen A, Valve R, Talja M, Fogelholm M: **Health psychological theory in promoting population health in Paijat-Hame, Finland: First steps towards a type 2 diabetes prevention study.** *J Health Psychol* 2004, **9:**73-84.

7. Schwarzer R: **Self-efficacy in the adoption and maintenance of health behaviors: Theoretical approaches and a new model.** In *Self-efficacy: Thought control of action.* Edited by Schwarzer R. Washington DC: Hemisphere Publishing Corp; 1992.

8. Gillison FB, Greaves CJ, Stathi A, Ramsay R, Bennett P, Taylor G, Francis M, Chandler R: **"Waste the Waist": The development of an intervention to promote changes in diet and physical activity for people with high cardiovascular risk.** *Br J Health Psychol* 2012, **17:**327-345.

9. Davis M, Fox K, Hillsdon M, Coulson J, Sharp D, Stathi A, Thompson J: **Getting out and about in older adults: the nature of daily trips and their association with objectively assessed physical activity.** *Int J Behav Nutr Phy* 2011, **8:**116.

10. NHS Choices: **The eatwell plate.** http://www.nhs.uk/Livewell/Goodfood/Pages/eatwell-plate.aspx: UK Department of Health; 2013.

11. Health Do: **Start Active, Stay Active: A report on physical activity for health from the four home countries' Chief Medical Officers.** London; 2011.

12. Rollnick S, Mason P, Butler C: *Health Behaviour Change: A guide for practitioners.* Edinburgh: Churchill Livingstone; 1999.

13. Miller WR, Rollnick S: *Motivational interviewing: Preparing people for change (2nd ed).* New York: Guildford Press; 2002.

14. Lindström J, Neumann A, Sheppard K, Gilis-Januszewska A, Greaves CJ, Handke U, Pajunen P, Puhl S, Pölönen A, Rissanen A, et al: **Take Action To Prevent Diabetes: A toolkit for the prevention of type 2 diabetes in Europe.** *Horm Metab Res* 2010, **42:**S37-S55.

15. Greaves CJ, Reddy P, Sheppard K: **Supporting behaviour change for diabetes prevention.** In *Diabetes Prevention in Practice.* Edited by Schwarz P, Reddy P, Greaves CJ, Dunbar J, Schwarz J. Dresden: TUMAINI Institute for Prevention Management; 2010: 19-29

16. Greaves CJ: **Supporting behaviour change in general practice.** In *Practical Psychology in Diabetes Care.* Edited by Barnard K, Lloyd CE. London: Springer-Verlag; 2012.

**APPENDIX 1: Detail of behavior change techniques^1^ used in Waste the Waist intervention**

**Table 1: Full set of behaviour change techniques incorporated within Waste the Waist**

- Provide general encouragement
- Motivational interviewing (style of delivery)
- Provide information about behavior health link
- Provide information on consequences of behavior in general
- Provide opportunities for social comparison
- Plan social support or social change
- Provide information on the process of behavior change^2^
- Provide opportunities/support for self-assessment^2^
- Prompt intention formation
- Prompt barrier identification/problem solving
- Prompt specific goal setting (behavior)
- Set graded tasks
- Action planning
- Prompt self-monitoring of behavior
- Teach to use prompts or cues (reading food labels; choosing from menus)
- Provide instruction on how to perform the behavior
- Provide information on *where and when* to perform the behaviour
- Relapse prevention/coping planning
- Prompt self-talk
- Stress management
- Prompting focus on past success

*Providing general encouragement, opportunities for social comparison* and delivering intervention content in a style aligned with *motivational interviewing* was common to all sessions. Further details, broken down into sessions, are provided below:

**Session 1**

*In relation to physical activity and diet, and their influence on cardiovascular disease and diabetes:*

- Provide information about behavior health link.
- Provide information on consequences of behavior in general.
- Provide opportunities for social comparison.
- Plan social support or social change.
- Provide information on the process of behavior change*
- Provide opportunities/support for self-assessment*

**Session 2 (+ 1 week)**

- Provide information on the process of behavior change*

*In relation to physical activity:*

- Provide opportunities/support for self-assessment*
- Prompt intention formation.
- Prompt barrier identification/problem solving.
- Prompt specific goal setting (behavior).
- Set graded tasks.
- Action planning
- Prompt self-monitoring of behavior (provide pedometer).
- Plan social support or social change.

**Session 3 (+2 weeks)**

*In relation to physical activity:*

- Provide feedback on performance.
- Prompt review of behavioral goals.
- Prompt self-monitoring of behavior.

*In relation to diet*

- Provide opportunities/support for self-assessment*
- Prompt intention formation.
- Prompt barrier identification/problem solving.
- Prompt specific goal setting (behavior).
- Set graded tasks.
- Action planning
- Prompt self-monitoring of behavior.
- Plan social support or social change.

**Session 4 (+3 weeks)**

*In relation to diet and physical activity*

- Provide feedback on performance.
- Prompt review of behavioral goals.
- Prompt barrier identification/problem solving.
- Prompt specific goal setting (behavior).
- Prompt self-monitoring of behavior.
- Plan social support or social change.
- Teach to use prompts or cues (reading food labels; choosing from menus)
- Provide information on *where and when* to perform the behaviour (shopping and dietary choices)
- Provide instruction on how to perform the behavior;
  - How to substitute lower fat and calorie alternatives
  - Mindfulness techniques

**Session 5 (+5 weeks)**

- Prompt self-talk (introduce CBT techniques for stopping negative thinking)

*Standard content of this and future maintenance sessions:*

- Relapse prevention/coping planning
- Provide feedback on performance.
- Prompt review of behavioral goals.
- Prompt barrier identification/problem solving.
- Prompt specific goal setting (behavior).
- Prompt self-monitoring of behavior.
- Plan social support or social change.

**Session 6 (+7 weeks)**

*In addition to standard content:*

- Provide information (dietician visit)

**Session 7 (+12 weeks)**

*In addition to standard content:*

- Provide instruction on how to perform the behavior (muscle strengthening activities)

**Session 8 (+20 weeks)**

*Standard techniques only:*

**Session 9 (+28 weeks)**

*In addition to standard content:*

- Prompting focus on past success

Notes:

^1^ As defined in: Michie S, Ashford S, Sniehotta FF, Dombrowski SU, Bishop A, French DP: **A refined taxonomy of behaviour change techniques to help people change their physical activity and healthy eating behaviours: the CALO-RE taxonomy.** *Psychol Health* 2011, **26:**1479-1498.

^2^ Behaviour change techniques included within the intervention but not mapping clearly to any techniques listed in the CALO-RE taxonomy.

**APPENDIX 2: Session Content**

**Session 1:** **Developing motivation**

**Objectives:**

- **To introduce group members to one another, and promote *active patient involvement* and positive group interactions**
- **To promote Waste the waist participants’ understanding that they are at high risk of developing heart disease and/or diabetes and would benefit from making lifestyle changes**
- **To prepare participants for lifestyle change by helping them to understand *how* to make lasting behaviour change**

**Main Messages**

- Changing your diet and physical activity will reduce your risk of cardio-vascular disease and type 2 diabetes
- Small changes can make a big difference to your weight and your health
- If you follow this programme, you will learn how to manage your lifestyle to make and sustain changes for life.
- Aim for a lifestyle that is both healthy and enjoyable - make changes that you can live with!

**Pre session reading:** Facilitators are advised to be particularly familiar with the session plan and Powerpoint slideshow for Session 1 and the following Session Materials:

**Session materials needed:**

- Sheet 1.0: Register
- Sheet 1.1: How the group should work
- Sheet 1.2: Process of behaviour change.
- Sheet 1.3: Myth busting
- Sheet 1.4: Programme goals
- Sheet 1.5: Decisional balance sheet
- Sheet 1.6: Importance ruler
- Sheet 1.7: Social support sheet.
- Sheet 1.8: Physical activity diary
- Sheet 1.9: End of session checklist

**Additional materials:**

- Flipchart stand, paper pads, coloured flipchart pens (2-3 sets needed)
- Laptop and powerpoint projector, extension lead
- Pedometers and pens for participants
- Digital recorder
- 10-12 Participant Manuals – one each
- Expense claim sheets for participant travel expenses

|  | **Timings** |
| --- | --- |
| **Outline of topics and timings** |  |
| 1. Welcome and introduction | 10 min |
| 1. Working together: Group guidelines | 8 min |
| 1. Understanding behaviour change | 13 min |
| 1. Overview of CVD risk | 20 min |
| SHORT BREAK | 10 min |
| 1. Programme goals | 2 min |
| 1. Getting motivated | 20 min |
| 1. Preparing for lifestyle change: social support | 15 min |
| 1. Take Away Tasks | 5 min |
| 1. Reflection on today’s session | 1 min |
|  | *104 min* |

**Session 2:** **Getting Going**

**Objectives:**

- **To encourage positive group interactions and active participant engagement with the facilitator, with each other and with the programme.**
- **To exchange information about what constitutes an active lifestyle and how physical activity is linked to CV risk.**
- **To help participants assess their physical activity levels and compare them with the current physical activity recommendations.**
- **To make a first steps action plan for increasing physical activity including goals, a coping plan and a plan for social support.**
- **To further increase participants’ understanding of the process of behaviour change and the strategies the Waste the Waist programme uses to help them succeed in making changes in diet and physical activity.**
- **To increase perceived importance of making changes to physical activity**
- **To increase perceived confidence about making changes to physical activity**

**Main Messages**

- A physically active lifestyle can make a real difference to your risk of heart disease.
- Moderate activity is ideal, but any increase in physical activity is beneficial.
- There are many different ways that you can incorporate more physical activity in your daily routine.
- Aim for a lifestyle that is both healthy and enjoyable - Make changes that you can live with.

**Pre session reading:**

Facilitators are advised to be familiar with this session plan, the Powerpoint slideshow for Session 2 and the following Session Materials:

**Session materials needed:**

- Sheet 2.0: Sign-up sheet
- Sheet 1.2: Process of behaviour change
- Sheet 1.4: Programme goals
- Sheet 1.6: Importance ruler
- Sheet 1.7: Social support sheet
- Sheet 1.8: Physical activity diary
- Sheet 2.2: Physical activity guidelines
- Sheet 2.3: Decisional Balance worksheet
- Sheet 2.4: Confidence ruler
- Sheet 2.5: Action planning sheet (PA action plan and Example PA action plan)
- Sheet 2.6: Barriers informational sheet
- Sheet 2.7: Food diary (for take away task)
- Sheet 2.8: Pros and cons of getting more active checklist (for Lifestyle Coach only)
- Sheet 2.9: SMART goal setting
- Sheet 2.10: End of session checklist

**Additional materials:**

- Weighing scales
- Flipchart – stand, paper pads, coloured flipchart pens (2-3 sets needed)
- Laptop and powerpoint projector
- Pedometers for participants
- Digital recorder
- 1-2 Spare participant manuals
- Expense claim sheets for participant travel expenses

| **Outline of topics and timings** | **Time** |
| --- | --- |
|  |  |
| 1. Welcome, review session 1, feedback on last week’s social support task, state objectives of today’s session | 10 mins |
| 1. Overview of physical activity | 20 mins |
| 1. How much are you getting? | 10 mins |
| 1. Assess importance of physical activity | 10 mins |
| 1. Assess confidence | 10 mins |
| SHORT BREAK | 10 mins |
| 1. Action planning: Setting SMART goals | 15 mins |
| 1. Action Planning: Social support | 5 mins |
| 1. Action Planning: Relapse prevention | 10 mins |
| 1. Keeping Track | 3 mins |
| 1. Take Away Tasks | 2 mins |
| 1. Reflection on session 2 | 2 mins |
| 1. Overview of next session | 1 mins |
| 1. Weight measurement (optional) /Q&As | 10 mins |
|  | *108 mins (+10 weight measurement)* |

**Session 3:** **Healthy Eating**

**Objectives:**

- **To encourage positive group interactions and active participant engagement with the facilitator, with each other and with the programme.**
- **To exchange information about what constitutes a healthy diet (as defined by the Eat Well Plate) and how healthy eating is linked to CV risk.**
- **To help participants assess their dietary habits and compare them with the Eat Well Plate recommendations.**
- **To make a “first steps” action plan for healthier eating including goals, a coping plan and a plan for social support.**
- **To further increase participants’ understanding of the process of behaviour change and the strategies the Waste the Waste programme uses to help them succeed in making changes in diet and physical activity.**
- **To increase perceived importance of making changes to diet.**
- **To increase perceived confidence about making changes to diet.**

**Main Messages**

- Healthy eating can make a real difference to your weight and to your risk of heart disease.
- A healthy diet is one where you limit the amount of fat and saturated fat, limit the amount of sugary drinks and snacks, get plenty of fruit and veg and plenty of starchy foods (especially those that are wholegrain).
- Changing the balance of things on your plate, changing the type of foods you choose (e.g. lower fat or wholegrain options) and changing the way you cook food are three ways to make your diet healthier.
- Aim for a diet that is both healthy and enjoyable!

**Pre session reading:**

Facilitators are advised to be particularly familiar with this session plan, the Powerpoint slideshow for Session 3 and the following Session Materials:

**Session materials needed (Participant Manual):**

- Sheet 3.0: Register
- Sheet 1.6: Importance ruler – Healthy eating
- Sheet 1.7b: Social support checklist
- Sheet 2.4: Confidence ruler – Healthy eating
- Sheet 3.0: Session 3 sign up sheet
- Sheet 3.2: Healthy Eating Checklist
- Sheet 3.4: Diet MOT Part 1 Slides as a handout
- Sheet 3.5: Fat and Fibre Quiz for Take Away task:
- Sheet 3.9: Healthy Eating Action Plan
- Sheet 3.10 End of session checklist

**Additional materials (Facilitators):**

- Weighing scales
- Flipchart – stand, paper pads, coloured flipchart pens (2-3 sets needed)
- Laptop and powerpoint projector
- 6 rulers (or other dividers for the Eatwell task)
- 2-3 Spare Food diaries
- 1-2 spare pedometers
- Digital recorder
- 1-2 Spare participant manuals
- Expense claim sheets for participant travel expense

| **Outline of topics and timings** | **Time** |
| --- | --- |
|  |  |
| 1. Introduction: Welcome back and recap session 2; state objectives of today’s session; review progress with take away tasks (physical activity plans (briefly); walking pace) | 8 mins |
| 1. Overview of healthy eating | 15 mins |
| 1. Assess Importance of healthy eating | 5 mins |
| 1. Eating Well: What is a healthy balanced diet? | 15 mins |
| SHORT BREAK | 10 mins |
| 1. Diet MOT | 20 mins |
| 1. Action planning: Setting SMART goals and Keeping track | 10 mins |
| 1. Action Planning: Social support | 5 mins |
| 1. Action Planning: Assess Confidence and Coping plans | 10 mins |
| 1. Take Away tasks | 1 mins |
| 1. Reflection on session 3 | 1 |
| 1. Overview of next week | 1 |
| 1. Weight measurement (optional) | 10 mins |
|  | *101 mins (+10 weight measure)* |

**Session 4: Dealing with temptations and challenges**

**Objectives:**

- **To encourage positive group interactions and active participant engagement with the facilitator, with each other and with the programme.**
- **To reinforce messages about what healthy eating is**
- **To practise food label reading and discuss ways to choose healthier alternatives when shopping.**
- **To discuss the temptations and challenges that can put our healthy eating action plans at risk when we eat out and to identify coping strategies.**
- **To discuss the link between food and mood**
- **To review progress with and update the “first steps” action plan for healthier eating.**
- **To review progress with and update the “first steps” action plan for physical activity.**
- **To further increase participants’ understanding of the process of behaviour change and the strategies the Waste the Waste programme uses to help them succeed in making changes in diet and physical activity.**

**Main Messages**

- Changing the balance of things on your plate, changing the type of foods you choose (e.g. lower fat or wholegrain options) and changing the way you cook food are three ways to make your diet healthier and to reduce the overall amount of calories you eat.
- Small changes can make a big difference
- Ways to control your appetite might include recognising and challenging automatic eating when it occurs – this involves putting up a mental STOP sign and trying to think of the healthy option in each situation.
- Finding alternative ways to manage stress or moods might also help you get better control over your appetite.
- Make changes that you can live with – aim for a lifestyle that is healthy and enjoyable.

**Pre session reading:**

Facilitators are advised to be particularly familiar with this session plan, the Powerpoint slideshow for Session 3 and the following Session Materials:

**Session materials needed:**

- Sheet 1.4: Programme goals
- Sheet 1.6: Importance ruler
- Sheet 2.3: Decisional balance sheet
- Sheet 2.4: Confidence ruler
- Sheet 2.7: Spare Food diaries
- Sheet 3.5: Fat and Fibre Quiz
- Sheets 3.9: Healthy Eating Action Plan
- Sheet 4.0: Sign up sheet
- Sheet 4.1 and 4.1a: Healthy swaps worksheet
- Sheet 4.2: Food Label Reading
- Sheet 4.3: Managing Food Cravings
- Sheet 4.4: Mindfulness
- Sheet 4.5: Small Changes
- Sheet 4.6: End of session checklist

**Additional materials:**

- **Shopping bag with 15-20 items (or food labels)**
- Weighing scales
- Flipchart – stand, paper pads, coloured flipchart pens (2-3 sets needed)
- Laptop and powerpoint projector
- 6 rulers (or other dividers for the Eatwell task)
- 1-2 spare pedometers
- 2-3 spare healthy eating action plans
- Digital recorder
- 1-2 Spare participant manuals
- Expense claim sheets for participant travel expenses

| **Outline of topics and timings** | **Time** |
| --- | --- |
|  |  |
| 1. Welcome, recap session 3, state objectives of today’s session | 2 min |
| 1. Review and update of physical activity action plans | 15 min |
| 1. The Fat and Fibre quiz – another tool for checking your diet | 10 min |
| 1. Label reading and shopping bag: What do we put in our basket? | 10 min |
| 1. Small changes make a big difference (and recipe modification) | 10 min |
| SHORT BREAK | 10 min |
| 1. Eating out: Social influences | 10min |
| 1. Managing Food Cravings | 10min |
| 1. Update healthy eating action plans | 20 min |
| 1. Homework | 2 min |
| 1. Reflection on session 4 | 1 min |
| 1. Overview of next week | 2 min |
| 1. Weight measurement (optional) with Q&A time in parallel | 10 min |
|  | *90 min (+10 for weighing)* |

**Session 5: Maintaining Motivation**

**Objectives: In this session, the participant will**

- **Review their progress with their existing ‘first steps’ action plans**
- **Identify any barriers to change and ways to overcome these barriers**
- **Review their motivations for making lifestyle changes**
- **Review their goals and consider adding to them (working towards longer-term goals)**
- **Review their efforts at engaging social support**
- **Revise their action plans accordingly**

**Main Messages**

- Aim for a lifestyle that is both healthy and enjoyable
- Make changes that you can live with
- Keep track of your progress;
  - using self-monitoring tools (like diaries or your pedometer) will help you to make sure you’re maintaining changes in your lifestyle, and not slipping back to old ways
  - comparing progress against your long-term targets will help you see whether the goals you’ve chosen are working for you
- Tackle set-backs head on - if things aren’t going well, take stock and try out different coping strategies until you find what works for you
- Challenge negative thinking patterns

**Pre session reading:**

Facilitators are advised to be particularly familiar with this session plan and the Powerpoint slideshow. It may also be useful to refresh your memory of action planning from its first introduction in Session 2.

**Session materials needed:**

- Sheet 5.0: Register
- Sheet 5.1: Progress review chart
- Sheet 5.2: Checklist for promoting enjoyment
- Sheet 5.4: End of session checklist

**Additional materials:**

- Weighing scales
- Fresh Action Plans for both diet and physical activity
- Laptop and projector for Powerpoint presentation
- 2-3 Pedometers for participants (to replace any lost)
- Flipchart – stand, paper pads, coloured flipchart pens (2-3 sets needed)
- Digital recorder
- 1-2 Spare participant manuals
- Expense claim sheets for participant travel expenses

| **Outline of topics and timings** | | **Time** |
| --- | --- | --- |
| (Pre-session: 10 min catch-up for those who missed last session) | | |
| 1. Welcome, briefly review key messages from session 4 | 5 mins | |
| 1. Review progress with diet and PA, celebrate success, re-frame failure | 15 mins | |
| 1. Problem solving & coping strategies | 20 mins | |
| 1. Positive thinking | 15 mins | |
| SHORT BREAK | 10 min | |
| 1. Revising action plans | 18 mins | |
| 1. Re-visiting self-monitoring | 4 mins | |
| 1. Take away tasks | 2 mins | |
| 1. Overview of next session | 2 mins | |
| 1. Weight measurement (optional) with Q&A time in parallel | 10 mins | |
| Total: | *91 (+10 min optional)* | |

**Session 6: Keeping Going (I)**

**Objectives: In this session, the participant will**

- **Review their progress with their existing diet and physical activity plans, celebrate success and reframe failure**
- **Review their motivations for making lifestyle changes (and amend action plans)**
- **Have an opportunity to discuss healthy eating with a dietitian**
- **Reinforce the importance of self-monitoring**
- **Review their goals and consider adding to them (working towards longer-term goals)**
- **Revise their action plans accordingly**

**Main Messages**

- Make changes that you can live with
- Keep track of your progress
  - Using self-monitoring tools (like diaries or the pedometer) will help you to make sure you’re maintaining changes in your lifestyle, and not slipping back to old ways
  - Comparing progress against your long-term targets will help you see whether the goals you’ve chosen are working for you
- Tackle set-backs head on - if things aren’t going well, take stock and try out different coping strategies until you find what works for you.

**Pre session reading:**

Facilitators are advised to be particularly familiar with this session plan, the Powerpoint slideshow for Session 6 and the following Session Materials:

**Session materials needed:**

- Sheet 6.0: Register
- Sheet 6.4: End of session checklist

**Additional materials:**

- Weighing scales
- Fresh Action Plans for both diet and physical activity
- Laptop and projector for Powerpoint presentation
- 2-3 Pedometers for participants (to replace any lost)
- Flipchart – stand, paper pads, coloured flipchart pens (2-3 sets needed)
- Digital recorder
- 1-2 Spare participant manuals
- Expense claim sheets for participant travel expenses

| **Outline of topics and timings** | **Time** | |
| --- | --- | --- |
| (Pre-session: 10 min catch-up for those who missed last session) | | |
| 1. Welcome, briefly review key messages from session 5 | | 4 mins |
| 1. Review progress with diet and PA, celebrate success, re-frame failure | | 15 mins |
| 1. Meet the Expert! Discussion about healthy eating | | 40 mins |
| SHORT BREAK | | 10 min |
| 1. Problem solving & coping strategies | | 20 mins |
| 1. Revising action plans | | 15 mins |
| 1. Revisiting self-monitoring | | 4 mins |
| 1. Take away tasks | | 1 min |
| 1. Overview of next session | | 1 min |
| 1. Weight measurement (optional) with Q&A time in parallel | | 10 mins |
| Total: | | *110*  *(+10 min optional)* |

**Session 7: Getting Stronger**

**Objectives: In this session, the participant will**

- **Review their progress with their existing diet and physical activity plans, celebrate success and reframe failure**
- **Review their motivations for making lifestyle changes**
- **Learn about and have an opportunity to discuss ideas about muscle strength training**
- **Reinforce the importance of self-monitoring**
- **Review their goals and consider adding to them (working towards longer-term goals)**
- **Revise their action plans accordingly**

**Main Messages**

- Adding muscle strength exercises to your physical activity plan could have some strong benefits, including reducing your risk of heart disease, strokes and type 2 diabetes.
- Aim for a lifestyle that is both healthy and enjoyable
- Make ‘sustainable’ changes: ones that you can live with
- Keep track of your progress
- Tackle set-backs head on - if things aren’t going well, take stock and try out different coping strategies until you find what works for you.

**Pre session reading:** Facilitators are advised to be particularly familiar with this session plan, the Powerpoint slideshow for Session 7 and the following Session Materials:

**Session materials needed:**

- Sheet 7.0: Register
- Sheet 5.1: Progress review chart (in User Manual)
- Sheet 7.1: Working your muscles (in User Manual)
- Sheet 7.4: End of session checklist

**Additional materials:**

- Weighing scales
- Fresh Action Plans for both diet and physical activity
- Laptop and projector for Powerpoint presentation
- 2-3 Pedometers for participants (to replace any lost)
- Flipchart – stand, paper pads, coloured flipchart pens (2-3 sets needed)
- Digital recorder
- 1-2 Spare participant manuals
- Expense claim sheets for participant travel expenses

| **Outline of topics and timings** | **Time** | |
| --- | --- | --- |
| (Pre-session: 10 min catch-up for those who missed last session) | | |
| 1. Welcome, briefly review key messages from session 6 | | 4 mins |
| 1. Review progress with diet and PA, celebrate success, re-frame failure | | 15 mins |
| 1. Getting Stronger: Muscle exercises and heart disease risk | | 30 mins |
| SHORT BREAK | | 10 min |
| 1. Are we having fun yet? | | 10 mins |
| 1. Problem solving & coping strategies | | 20 mins |
| 1. Revising action plans | | 15 mins |
| 1. Revisiting self-monitoring | | 4 mins |
| 1. Take away tasks | | 1 min |
| 1. Overview of next session | | 1 min |
| 1. Weight measurement (optional) with Q&A time in parallel | | 10 mins |
| Total: | | *110*  *(+10 min optional)* |

**Session 8: Keeping Going**

**Objectives: In this session, the participant will**

- **Review their progress with their existing diet and physical activity plans, celebrate success and reframe failure**
- **Discuss ways for managing stress**
- **Review their motivations for making lifestyle changes**
- **Review their goals and consider adding to them (working towards longer-term goals)**
- **Revise their action plans accordingly**

**Main Messages**

- Make ‘sustainable’ changes: ones that you can live with
- Keep track of your progress
- Tackle set-backs - if things aren’t going well, take stock and try out different coping strategies until you find what works for you.
- You can use simple techniques to manage stressful events and situations.

**Pre session reading:**

Facilitators are advised to be particularly familiar with this session plan, the Powerpoint slideshow for Session 8 and the following Session Materials:

**Session materials needed:**

- Sheet 8.0: Register
- Sheet 5.1: Progress review chart
- Sheet 8.2: Stress management
- Sheet 8.3: Progressive relaxation worksheet
- Sheet 8.4: End of session checklist,

**Additional materials:**

- Weighing scales
- Fresh Action Plans for both diet and physical activity
- Laptop and projector for Powerpoint presentation
- 2-3 Pedometers for participants (to replace any lost)
- Flipchart – stand, paper pads, coloured flipchart pens
- Digital recorder
- 1-2 Spare participant manuals
- Expense claim sheets for participant travel expenses

| **Outline of topics and timings** | **Time** | |
| --- | --- | --- |
| (Pre-session: 10 min catch-up for those who missed last session) | | |
| 1. Welcome, briefly review key messages from session 7 | | 4 mins |
| 1. Review progress with diet and PA, celebrate success, re-frame failure | | 15 mins |
| 1. Stress Management | | 30 mins |
| SHORT BREAK | | 10 min |
| 1. Problem solving & coping strategies | | 20 mins |
| 1. Revising action plans | | 20 mins |
| 1. Take away tasks | | 1 min |
| 1. Relaxation technique | | 10 mins |
| 1. Overview of next session | | 1 min |
| 1. Weight measurement (optional) with Q&A time in parallel | | 10 mins |
| Total: | | 111  (+10 min optional) |

**Session 9: Into the Future**

**Objectives: In this session, the participant will**

- **Review their progress in using the general process for making lifestyle changes**
- **Review their progress with their existing diet and physical activity plans (celebrate any successes and reframe failures)**
- **Review their motivations for making lifestyle changes**
- **Think about the longer term and their “possible future selves”**
- **Review their goals and consider adding to them (working towards longer-term goals)**
- **Discuss ways of maintaining weight loss and taking it further**

**Main Messages**

- Aim for a lifestyle that is both healthy and enjoyable
- Make changes that you can live with
- Keep track of your progress
- Try out different coping strategies until you find what works for you
- If you want to go further, add some more changes

**Pre session reading:**

Facilitators are advised to be particularly familiar with this session plan, the Powerpoint slideshow for Session 9 and the following Session Materials:

**Session materials needed:**

- Sheet 9.0: Register
- Sheet 5.1: Progress review chart
- Sheet 9.2a, 9.2b: Eating Out
- Sheet 9.4: End of session checklist

**Additional materials:**

- Weighing scales
- Fresh Action Plans for diet and physical activity
- Laptop and projector for Powerpoint presentation
- 2-3 Pedometers for participants (to replace any lost)
- Flipchart – stand, paper pads, coloured flipchart pens
- Digital recorder
- 1-2 Spare participant manuals
- Expense claim sheets for participant travel expenses

| **Contents** | **Time** | |
| --- | --- | --- |
| (Pre-session: 10 min catch-up for those who missed last session) | | |
| 1. Welcome, briefly review key messages from session 8 | | 2 mins |
| 1. Review progress with diet and PA, celebrate success, re-frame failure. 2. Problem solving & coping strategies | | 10 mins  10 mins |
| 1. Review progress with using the process of lifestyle change | | 10 mins |
| 1. Tailing off /keeping the ball rolling | | 15 mins |
| SHORT BREAK | | 10 min |
| 1. Choice of activities: Eating out or client-led activity /open discussion | | 15 mins |
| 1. Maintaining lifestyle changes | | 15 mins |
| 1. Revising action plans | | 15 mins |
| 1. Take away tasks and final farewell | | 2 mins |
| 1. Weight measurement (optional) with Q&A time in parallel | | 10 mins |
| Total: | | *104*  *(+10 min optional)* |
