# Supplementary material for: Processes of behavior change and weight loss in a theory-based weight loss intervention program: a test of the process model for lifestyle behavior change
Source: Int J Behav Nutr Phys Act. 2015 Jan 16;12:2. doi: 10.1186/s12966-014-0160-6 (PMC4304200; doi:10.1186/s12966-014-0160-6)
Supplement: Additional file 3: — Selection of Measures for Waste the Waist Evaluation. [file 12966_2014_160_MOESM3_ESM.docx]

**Selection of Measures for Waste the Waist Evaluation**

**Criteria for selection of measures**

Measures were selected /adapted to meet the following criteria:

1. Brevity (ideally 4 items or less)
2. Good evidence of construct validity and internal reliability
3. Demonstrated sensitivity to change in a dietary or physical activity intervention setting.

Where optimal measures could not be found, we constructed brief items ourselves based on concepts that are directly targeted by the intervention in order to maximise sensitivity to change. This was a limitation of the study, but was considered necessary to allow exploration of the proposed concept in some form, and to develop and refine measures of relevant concepts for future use in this field.

Where there were good measures that were too long, we looked for the 4 highest-loading items (highest item-total correlation) using our existing datasets, but also moderated our selection by considering which items were most relevant to the processes /content of our own intervention.

| **Process** | **Measures** | **Timeframe** |
| --- | --- | --- |
| 1. *Understanding the process of behaviour change* | Brief questionnaire, constructed by C Greaves and piloted /refined with feedback from 15 people: Covers knowledge about how to make permanent changes to behaviour, how to get and stay motivated, the perceived importance of social support, knowing how to overcome barriers and having skills to manage food cravings. | 0, 4, 12 months |
| 1. *Perceived Importance* | Perceived importance of eating a healthy diet and doing at least 150 mins /wk of MVPA (with a brief definition provided).  We have used a 0 to 10 VAS, alongside the adapted the Intrinsic Motivation Inventory [1] by reducing the number of items to 4 and providing 3 specific intrinsic motivations that should be relevant to the target group and /or which are targeted by the intervention (helping to control my weight; reducing my risk of getting heart disease; contributing to my sense of well-being). This had been found to be applicable to adult populations trying to lose weight [2]. | 0, 4, 12 months and interim within-session measures (VAS taken at the point of action-planning)  0, 3, 12 months and interim within-session measures |
| 1. *Explore and enhance motivation for change (Self Efficacy and confidence)* | SE for healthy eating: We selected a 5-item reduced /modified version of the Weight Efficacy Life-Style Questionnaire, developed by Linde, Rothman, Baldwin and Jeffery [3]. We have also used a 0 to 10 VAS scale to assess confidence about eating a healthy diet (definition provided) over a) the next month and b) the next 12 months  Self efficacy for healthy level of PA: We selected a 5-item scale developed by Marcus, Selby, Niaura and Rossi [4]. We have also used a 0 to 10 VAS scale to assess confidence about being able to achieve a healthy level of physical activity (definition provided) over a) the next month and b) the next 12 months. | 0, 4, 12 months and interim within-session measures  0, 4, 12 months and interim within-session measures |
| 1. *Identify and engage sources of social support* | Social support for healthy diet: We have selected a 5-item adaptation of the Sallis et al [5] scale refined by Norman et al [6].  Social support for healthy level PA: We have selected a 5-item adaptation of the Sallis et al [5] scale refined by Roesch et al [7]. | 0, 4, 12 months  0, 4, 12 months |
| 1. *Action planning* | Level of engagement with action planning process.  1. From coding of completed action plans (design to be described by XX, University of Norwich, as part of her PhD, but to indicate level of engagement with the 3 elements of goal-setting, coping planning and social support planning, as well as participant (later) ratings of how useful the plans / reviewing of plans were).  2. We used 4 items on action planning and 3 items on coping planning from the scale developed by Sniehotta et al [8]. The item removed was on “with whom to exercise” as the factor loadings were split for this item. | 0, 4, 12 months (and data from ongoing collection of action plans) |
| 1. *Diet* | The DINE measure, which has been validated for use by non-dietician specialists in primary care was selected [9]. | 0, 4, 12 months |
| 1. *Self-regulation* | Frequency /regularity of self-monitoring and relapse management assessed using 2 items on self-monitoring from Sniehotta, Scholz and Schwarzer [10], 2 newly constructed items on attempts to identify and solve problems and learn from experience, and 1 (new) item on general salience of physical activity, all over the last month.  Following Linde, Jeffery, Finch, Ng and Rothman [11], we also asked “During the past week, on how many days did you weigh yourself?”. | 0, 4, 12 months |
| Additional Processes |  |  |
| 1. *Autonomy support.* | We included items from the Learning Climate Questionnaire [12] in the client satisfaction questionnaire (for Intervention group only) to assess how autonomy support participants felt was provided by their lifestyle coaches. | 4, 12 months |
| 1. *Quality of intra-group interactions* | Assessed through the Physical Activity Group Environment Questionnaire [13]. The 6 items which seem to have the most face validity for this intervention were selected. | 4, 12 months |
| 1. *Affective responses* | Affective response to PA: We selected 4 items from an 8-item short version of the Physical Activity Enjoyment Scale (PACES) [14]. Items were selected to represent diversity (several items in the 8-item scale simply use different words for ‘enjoy’). The scale asks “how you feel when you are doing moderately intense physical activity” (and we provide a simple definition of moderate activity).  Affective evaluation of eating a healthy diet. We selected 2 items from the Interest /Enjoyment scale of the Intrinsic Motivation Inventory [15] asking about enjoyment of “healthy foods” (with a definition provided) and added an item asking for level of agreement with the statement “I have found a diet that is both healthy and enjoyable”.  For impulse control /managing food cravings, we selected 10 items from the 18-item version of the Three Factor Eating Questionnaire [16]. The selected items represent the sub-scales for ‘cognitive restraint’ (all 6 items) and ‘uncontrolled eating’ (4 of 9 items). | 0, 4, 12 months |

Notes: MVPA Moderate to vigorous physical activity; VAS visual analogue scale; SE self-efficacy; PA physical activity

**References**

1. McAuley E, Duncan T, Tammen VV: **Psychometric properties of the intrinsic motivaiton inventory in a competitive sport setting: A confirmatory factor analysis.** *Research Quarterly for Exercise and Sport* 1989, **60:**48-58.

2. Teixeira PJ, Going SB, Houtkooper LB, Cussler EC, Metcalfe LL, Blew RM, Sardinha LB, Lohman TG: **Exercise motivation, eating, and body image variables as predictors of weight control.** *Med Sci Sports Exerc* 2006, **38:**179-188.

3. Linde JA, Rothman AJ, Baldwin AS, Jeffery RW: **The impact of self-efficacy on behavior change and weight change among overweight participants in a weight loss trial.** *Health Psychol* 2006, **25:**282-291.

4. Marcus BH, Selby VC, Niaura RS, Rossi JS: **Self-efficacy and the stages of exercise behavior change.** *Res Q Exerc Sport* 1992, **63:**60-66.

5. Sallis JF, Grossman RM, Pinski RB, Patterson TL, Nader PR: **The development of scales to measure social support for diet and exercise behaviors.** *Prev Med* 1987, **16:**825-836.

6. Norman GJ, Carlson JA, Sallis JF, Wagner N, Calfas KJ, Patrick K: **Reliability and validity of brief psychosocial measures related to dietary behaviors.** *Int J Behav Nutr Phys Act* 2010, **7:**56.

7. Roesch SC, Norman GJ, Villodas F, Sallis JF, Patrick K: **Intervention-mediated effects for adult physical activity: A latent growth curve analysis.** *Soc Sci Med* 2010, **71:**494-501.

8. Sniehotta FF, Schwarzer R, Scholz U, Schüz B: **Action planning and coping planning for long-term lifestyle change: theory and assessment.** *European Journal of Social Psychology* 2005, **35:**565-576.

9. Roe L, Strong C, Whiteside C, Neil A, Mant D: **Dietary intervention in primary care: validity of the DINE method for diet assessment.** *Fam Pract* 1994, **11:**375-381.

10. Sniehotta FF, Scholz U, Schwarzer R: **Action plans and coping plans for physical exercise: A longitudinal intervention study in cardiac rehabilitation.** *Br J Health Psychol* 2006, **11:**23-37.

11. Linde JA, Jeffery RW, Finch EA, Ng DM, Rothman AJ: **Are unrealistic weight loss goals associated with outcomes for overweight women?** *Obes Res* 2004, **12:**569-576.

12. Williams GC, Deci EL: **Internalization of biopsychosocial values by medical students: a test of self-determination theory.** *J Pers Soc Psychol* 1996, **70:**767-779.

13. Estabrooks PA, Carron AV: **The Physical Activity Group Environment Questionnaire: An instrument for the assessment of cohesion in exercise classes. .** *Group Dynamics: Theory, Research, and Practice* 2000, **4:**230-243.

14. Kendzierski D, DeCarlo KJ: **Physical activity enjoyment scale: Two validation studies.** *Journal of Sport & Exercise Psychology* 1991, **13:**50-64.

15. McAuley E, Duncan T, Tammen VV: **Psychometric properties of the Intrinsic Motivation Inventory in a competitive sport setting: a confirmatory factor analysis.** *Res Q Exerc Sport* 1989, **60:**48-58.

16. de Lauzon B, Romon M, Deschamps V, Lafay L, Borys JM, Karlsson J, Ducimetiere P, Charles MA, Fleurbaix Laventie Ville Sante Study G: **The Three-Factor Eating Questionnaire-R18 is able to distinguish among different eating patterns in a general population.** *J Nutr* 2004, **134:**2372-2380.
