# Supplementary material for: Processes of behavior change and weight loss in a theory-based weight loss intervention program: a test of the process model for lifestyle behavior change
Source: Int J Behav Nutr Phys Act. 2015 Jan 16;12:2. doi: 10.1186/s12966-014-0160-6 (PMC4304200; doi:10.1186/s12966-014-0160-6)
Supplement: Additional file 4: Tables S1 and S2. — Distribution and psychometric properties of study variables at baseline. [file 12966_2014_160_MOESM4_ESM.docx]

Supplementary Table S1: Distribution and psychometric properties of study variables at baseline

| Measure | N | Minimum | Maximum | Mean | Std. Deviation | Skewness | Kurtosis | Cronbach α |
| --- | --- | --- | --- | --- | --- | --- | --- | --- |
| *Outcome measures* | |  |  |  |  |  |  |  |
| Weight loss 4 months (kg) | 96 | -14.60 | 6.65 | -2.14 | 3.70 | -0.53 | 0.99 | - |
| Weight loss 1 yr (kg) | 96 | -26.50^c^ | 10.40 | -3.10 | 6.31 | -1.05 | 1.63 | - |
| Fiber intake (g) | 106 | 12.00 | 60.00 | 36.60 | 10.60 | 0.11 | -0.28 | - |
| Fat intake (g) | 106 | 11.00 | 58.00 | 31.08 | 10.01 | 0.31 | -0.11 | - |
| MVPA^b^ | 106 | .4 | 94.0 | 22.02 | 19.53 | - | - | - |
| Overall PA count^b^ | 106 | 53.41 | 633.72 | 255.56 | 113.13 | - | - | - |
| *Process measures* |  |  |  |  |  |  |  |  |
| Understanding process | 99 | 1.67 | 4.83 | 3.23 | 0.65 | -0.61 | 0.12 | 0.82 ^a^ |
| Perceived importance |  |  |  |  |  |  |  |  |
| Diet – composite score | 100 | 5.00 | 7.00 | 6.44 | 0.61 | -0.64 | -0.89 | 0.70 |
| PA – composite score | 100 | 3.50 | 7.00 | 6.32 | 0.76 | -1.24 | 1.61 | 0.82 |
| Diet - VAS | 74 | 5.00 | 10.00 | 8.64 | 1.35 | -0.78 | 0.17 |  |
| PA - VAS | 71 | 3.00 | 10.00 | 7.97 | 1.83 | -0.61 | -0.12 |  |
| Self-efficacy diet |  |  |  |  |  |  |  |  |
| Diet | 100 | 1.00 | 8.00 | 4.43 | 1.56 | -0.01 | -0.48 | 0.87 |
| PA | 100 | 1.00 | 5.00 | 2.85 | 0.91 | -0.24 | -0.28 | 0.87 |
| Social support |  |  |  |  |  |  |  |  |
| Diet | 100 | 1.67 | 5.00 | 3.20 | 0.83 | -0.04 | -0.82 | 0.81 |
| PA | 100 | 1.00 | 5.00 | 2.21 | 1.04 | 0.59 | -0.68 | 0.91 |
| Action planning |  |  |  |  |  |  |  |  |
| Diet | 98 | 1.00 | 4.00 | 2.78 | 0.75 | -0.55 | 0.14 | 0.92 |
| PA | 99 | 1.00 | 4.00 | 2.47 | 0.79 | -0.35 | -0.42 | 0.95 |
| Coping planning |  |  |  |  |  |  |  |  |
| Diet | 98 | 1.00 | 4.00 | 2.39 | 0.72 | -0.15 | -0.40 | 0.94 |
| PA | 98 | 1.00 | 4.00 | 2.16 | 0.76 | 0.03 | -0.48 | 0.91 |
| Self-monitoring |  |  |  |  |  |  |  |  |
| Diet | 99 | 1.20 | 4.00 | 2.56 | 0.54 | -0.03 | 0.46 | 0.73^a^ |
| PA | 99 | 1.00 | 3.80 | 2.34 | 0.56 | 0.07 | -0.33 | 0.70 ^a^ |
| Enjoyment |  |  |  |  |  |  |  |  |
| Diet | 100 | 1.67 | 7.00 | 4.78 | 1.31 | -0.15 | -0.60 | 0.71 |
| PA | 99 | 1.00 | 7.00 | 3.85 | 1.42 | -0.01 | -0.37 | 0.90 |
| Cognitive restraint | 99 | 1.17 | 3.67 | 2.29 | 0.52 | 0.21 | -0.33 | 0.68 |
| Uncontrolled eating | 99 | 1.00 | 3.50 | 1.99 | 0.69 | 0.27 | -0.85 | 0.78 |

Notes: ^a^  indicates value calculated after items deleted, as detailed in Methods section; ^b^ Only participants reporting ≥ 4 days complete accelerometry data at each time point included; ^c^ This significant weight loss was achieved by a participant allocated to the control group who had joined the program shortly after being widowed, and had embraced the program as part of a set of significant, wider lifestyle changes.

Supplementary Table S2a: Correlations between change scores for dietary self-regulatory behaviors and change in dietary motivational influences and dietary behaviors

|  | N | Study outcome variables | | | Motivation-related processes | | | |  |
| --- | --- | --- | --- | --- | --- | --- | --- | --- | --- |
|  |  | Weight | Fat intake^±^ | Fiber intake^±±^ | Understanding | Perceived importance | Self-efficacy | Social support | R^2^ (df=3,47)^a^ |
| *4 months* |  |  |  |  |  |  |  |  |  |
| Action planning | 85 | -0.30 | -0.16 | 0.11 | 0.14 | 0.15 | 0.35** | 0.29** | 0.18 NS |
| Coping planning | 85 | -0.12 | -0.17 | 0.08 | 0.16 | 0.18 | 0.45*** | 0.20 | 0.20* |
| Self-monitoring | 86 | -0.23* | -0.21 | 0.09 | 0.39*** | 0.27 | 0.43*** | 0.33** | 0.42***^c^ |
| *12 months* |  |  |  |  |  |  |  |  |  |
| Action planning | 83 | -0.13 | -0.08 | 0.07 | 0.17 | 0.11 | 0.20 | 0.43*** | 0.09 NS |
| Coping planning | 83 | -0.23* | -0.22 | -0.03 | 0.19 | 0.21 | 0.28* | 0.34** | 0.17* |
| Self-monitoring | 83 | -0.18 | -0.25* | 0.10 | 0.39*** | 0.15 | 0.53*** | 0.13 | 0.39***^bd^ |

^a^ R^2^ refers to the regression model predicting self-regulatory behavior from the four motivation-related process variables, ^b^ Significant predictor = self-efficacy, ^c^ Significant predictor = social support, ^d^ Significant predictor = understanding; ^±^ N=82, ^±±^N=81; *p<.05, **p<.01, ***p<.001

Supplementary Table S2b: Correlations between change scores for physical activity self-regulatory behaviors and change in motivational influences, physical activity behavior and weight

|  | N | Study outcome variables | | Motivation-related processes | | |  |  |
| --- | --- | --- | --- | --- | --- | --- | --- | --- |
|  |  | Weight | MVPA^±^ | Understanding | Perceived importance | Self-efficacy | Social support | R^2^ (df=3,47)^a^ |
| *4 months* |  |  |  |  |  |  |  |  |
| Action planning | 84 | -0.00 | -0.12 | 0.15 | 0.35* | 0.35* | 0.29** | 0.32**^c^ |
| Coping planning | 84 | -0.15 | 0.08 | 0.14 | 0.43** | 0.26* | 0.20 | 0.30**^ce^ |
| Self-monitoring | 85 | -0.15 | -0.16 | 0.47*** | 0.56*** | 0.45*** | 0.33** | 0.57***^bd^ |
| *12 months* |  |  |  |  |  |  |  |  |
| Action planning | 83 | -0.08 | 0.27* | 0.24* | 0.19 | 0.37** | 0.43*** | 0.09, NS |
| Coping planning | 82 | -0.17 | 0.29* | 0.28* | 0.14 | 0.34** | 0.34** | 0.17*^e^ |
| Self-monitoring | 83 | 0.10 | 0.05 | 0.41*** | 0.11 | 0.40*** | 0.13 | 0.39***^bd^ |

^a^ R^2^ refers to the regression model predicting self-regulatory behavior from the four motivation-related process variables, ^b^ Significant predictor = self-efficacy, ^c^ Significant predictor = social support, ^d^ Significant predictor = understanding, ^e^ Significant predictor = perceived importance; ^±^N=76; *p<.05, **p<.01, ***p<.001.
